# A cross-sectional evaluation of large language model chatbot interfaces for patient-facing herpes zoster information: safety, information quality, and readability

**DOI:** 10.3389/fpubh.2026.1955701

**Published:** 2026-09-16

**Authors:** Dalian Liang, Cong Mai, Zhengkun Zhang, Jieyun Lin, Xianmei Wu, Haiying Lu, Jingli Shang, Huajun Wang, Haiyan Li

**Affiliations:** 1 Department of Critical Care Medicine, Guangdong Provincial People’s Hospital (Guangdong Academy of Medical Sciences), Southern Medical University, Guangzhou, Guangdong, China; 2 Department of Cardiac Intensive Care, Guangdong Province Maoming People's Hospital, Maoming, Guangdong, China

**Keywords:** chatbots, herpes zoster, information quality, large language models, patient education, readability, safety, *Shingles*

## Abstract

**Background:**

Large language model (LLM) chatbots are increasingly used to obtain health information. However, fluent and clinically plausible responses may still contain safety-relevant omissions, inadequate source attribution and disclosure, or difficult-to-read text.

**Objective:**

To evaluate the safety, accuracy, empathy, information quality, response-level source attribution and disclosure, overall quality, and readability of five LLM chatbot interfaces answering lay-oriented questions about herpes zoster.

**Methods:**

This cross-sectional evaluation used 46 standardised English-language questions across seven herpes zoster domains. Each question was submitted once to ChatGPT-5.5 Instant, DeepSeek-V4-Pro, Doubao-Seed-2.0-Pro, Gemini 3.5 Flash, and Qwen3.7-Plus under consumer-access conditions on June 2–3, 2026. Five dermatologists independently assessed safety, accuracy, empathy, DISCERN, Ensuring Quality Information for Patients (EQIP), Journal of the American Medical Association (JAMA) benchmark criteria, and the Global Quality Score (GQS). Six readability indices were calculated. Paired comparisons and inter-rater agreement were evaluated using prespecified statistical methods.

**Results:**

The five interfaces generated 230 complete responses. Under the study conditions, 66 responses (28.7%) were classified as potentially unsafe using the prespecified ≥3/5-rater majority threshold, with no significant difference between interfaces (Cochran’s Q = 1.661, df = 4, *p* = 0.798). Sensitivity analyses using alternative thresholds showed consistent results. Accuracy and empathy differed across interfaces (both *p* < 0.001; Kendall’s *W* = 0.127 and 0.211, respectively), although effect sizes were small. Information-quality and overall-quality measures also showed outcome-specific differences. All six readability indices differed across interfaces (all *p* < 0.001); DeepSeek-V4-Pro generally produced text estimated to be easier to read, whereas Gemini 3.5 Flash produced text estimated to be more difficult to read. Safety agreement was substantial (Fleiss’ κ = 0.682), and ICC (2,1) values for other manually rated outcomes ranged from 0.832 to 0.889.

**Conclusion:**

In this standardised benchmark, LLM chatbot interfaces provided generally favourable accuracy and information-quality scores but showed clinically relevant safety limitations, sparse source attribution and disclosure, and readability challenges. No interface consistently performed best across all outcomes. These findings represent single first responses obtained under the tested conditions and do not establish response stability across repeated queries. LLM-generated herpes zoster information should therefore be interpreted cautiously and should not replace individualised professional assessment or professionally reviewed patient information.

## 1 Introduction

Herpes zoster (HZ) results from reactivation of latent varicella-zoster virus and disproportionately affects older and immunocompromised adults (1). After primary infection, the virus remains latent in dorsal-root or cranial-nerve ganglia (1, 2). Incidence rises sharply after 50 years of age (3). Lifetime risk is approximately 30% in the general population and may approach 50% among people who live to 85 years (2, 4). HZ typically causes a painful vesicular rash and can substantially impair quality of life (5, 6). Complications occur in up to 30% of cases. Postherpetic neuralgia is the most common complication and can cause persistent, debilitating pain (7, 8). Other complications include ocular and neurological disease and an increased long-term risk of cardiovascular events (9, 10).

Large language models (LLMs) are increasingly used in medical education, clinical documentation, decision support, patient communication, and health-information delivery (11). Artificial-intelligence methods have also been investigated for HZ diagnosis, including image-based applications (12, 13). Many patients seek health information online when professional resources are difficult to access (14). Consumer-facing chatbots such as ChatGPT, Gemini, DeepSeek, Doubao, and Qwen now provide immediate conversational answers to health questions. Survey data suggest that these tools are becoming an additional source of online health information, especially among younger users and people with favourable attitudes towards medical AI (15). Their interactive format may shift some information seeking from keyword-based searches towards personalised dialogue.

Fluent and clinically plausible language does not guarantee that an answer is accurate, complete, safe, or appropriate for an individual user. Medical LLMs have demonstrated substantial clinical knowledge in structured tasks (16). However, evaluations of clinical decision-making have identified factual errors, reasoning limitations, potential harm, and sensitivity to the order and framing of information (17). Outputs may also be inconsistent or deviate from clinical guidelines (17). These limitations are important for HZ because incorrect advice about urgent symptoms, antivirals, dosing, high-risk contacts, pregnancy, immunocompromise, or vaccination could delay care or alter behaviour. Evaluations should therefore distinguish factual accuracy from safety, empathy, information quality, source attribution and disclosure, overall quality, and readability.

Readability is a further concern. The American Medical Association recommends that patient-oriented materials be written at approximately a sixth-grade reading level (18). Studies across several clinical domains indicate that LLM-generated patient information often exceeds this threshold, although readability varies by platform and prompting strategy (19–21). Explicitly requesting sixth-grade language can improve readability without necessarily reducing DISCERN-rated quality (20). Readability should therefore be evaluated alongside clinical quality rather than treated as a secondary presentation feature.

Within dermatology, a growing body of research has evaluated LLM-generated health information and patient education across dermatologic conditions, examining dimensions such as accuracy, information quality, empathy, reliability, and readability (21–24). HZ-related research has also begun to emerge, including evaluations of dermatologic patient-education materials containing herpes zoster-related information and ChatGPT-generated guidance concerning recombinant zoster vaccination (21, 25). However, these studies have generally focused on specific educational formats or narrowly defined clinical topics. A broader multidimensional comparison of multiple consumer-facing chatbot interfaces across common and safety-critical HZ information needs remains limited.

Accordingly, the present study was designed as a multidimensional exploratory evaluation of five consumer-facing LLM chatbot interfaces using 46 clinician-selected, lay-oriented HZ questions intended to simulate public queries. Prespecified evaluation domains included safety, accuracy, empathy, information quality assessed using DISCERN and EQIP, response-level source attribution and disclosure assessed using the JAMA benchmark criteria, overall quality assessed using GQS, and textual readability assessed using six formula-based indices. We further examined whether generally favourable accuracy and information-quality assessments were accompanied by adequate safety safeguards, source attribution and disclosure, and readable text under standardised first-response conditions.

## 2 Methods

### 2.1 Study design and reporting framework

This cross-sectional health-information evaluation assessed the initial response that a lay user would receive from five publicly accessible LLM chatbot interfaces. It did not develop or validate a diagnostic algorithm, modify model weights, or assess patient outcomes. Reporting was guided by the Chatbot Assessment Reporting Tool (CHART) (26). The overall study workflow is shown in Figure 1.

**Figure 1:**
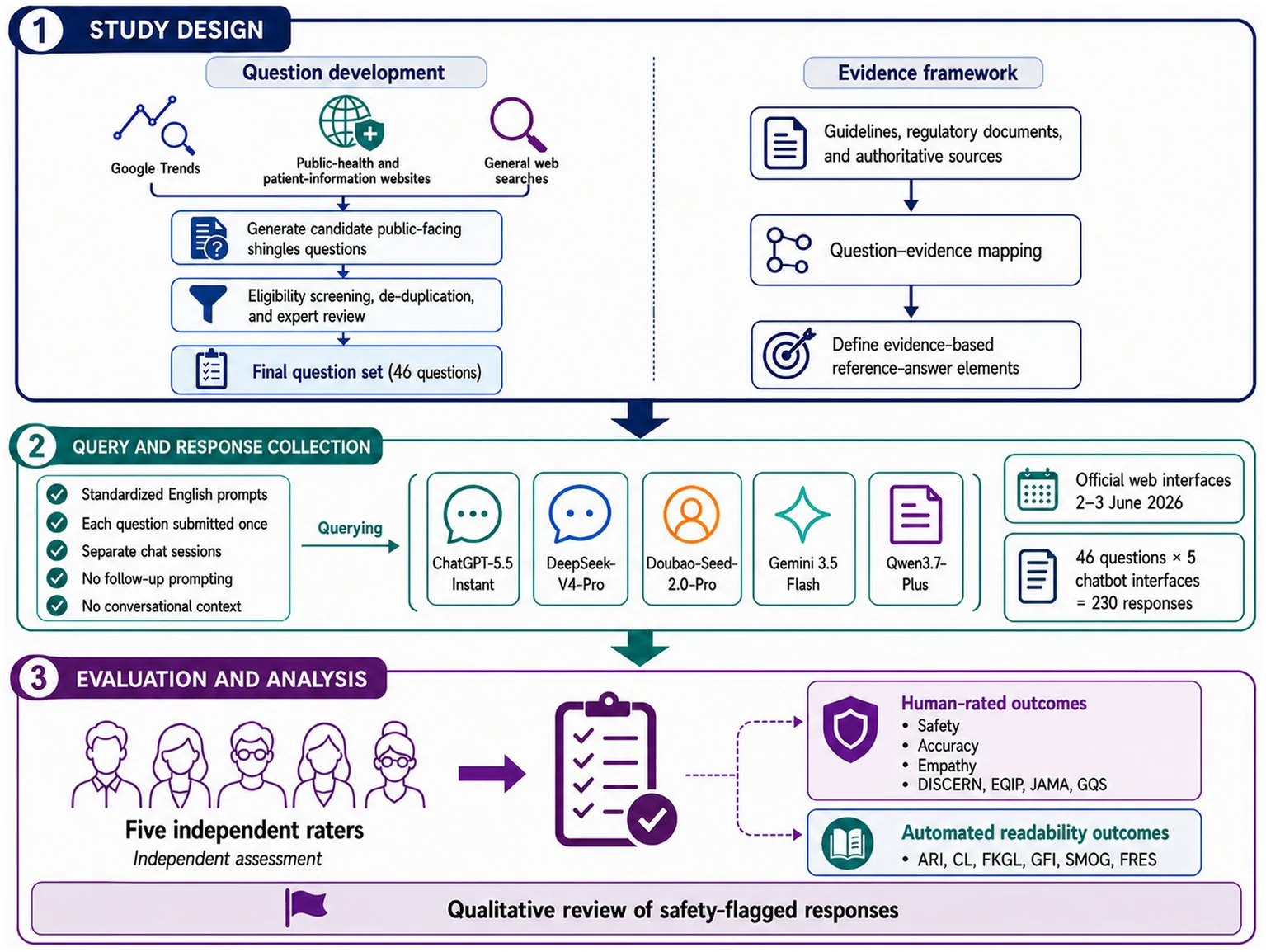
Study workflow. Question identification and screening were followed by chatbot querying, response capture, majority-based manual evaluation, readability assessment, and paired statistical analysis across five LLM chatbot interfaces.

### 2.2 Chatbot interface selection and access

Five publicly accessible, general-purpose LLM chatbot interfaces were evaluated: ChatGPT-5.5 Instant, DeepSeek-V4-Pro, Doubao-Seed-2.0-Pro, Gemini 3.5 Flash, and Qwen3.7-Plus. The displayed interface label was manually selected on each platform before data collection and recorded as interface metadata. Because all systems were accessed through consumer web interfaces, these labels should not be interpreted as verified API model identifiers or backend checkpoints. Displayed web-interface labels, official access URLs, access conditions, and reproducibility limitations for all five interfaces are summarised in Supplementary Table S1. We therefore use the term “interface” throughout the manuscript. All interfaces were accessed manually through official provider-hosted consumer websites using free accounts. A single free consumer account was used for each interface throughout data collection, and all queries were conducted on the same computer using the same web browser. No application programming interfaces, automated scripts, browser extensions, plug-ins, or third-party aggregators were used. User-accessible memory and personalisation were disabled where available. Search or retrieval functions were neither manually activated nor disabled; each interface could invoke these functions according to its default deployed behaviour. No external documents, URLs, reference materials, or source lists were supplied. Data were collected on June 2–3, 2026, in Guangzhou, China (China Standard Time, UTC + 8). Each question was entered manually in a new conversation without chat history, follow-up prompting, regeneration, prompt optimisation, custom instructions, or selective replacement. The first complete response was retained. Generation parameters, including temperature, top-p, token limits, and random seeds, were neither displayed nor controllable in the consumer web interfaces. If a quota warning, model-switch notification, interruption, or incomplete response occurred, collection was paused until the selected interface became available. Responses generated after an automatic downgrade were excluded. The exact backend checkpoints, release or last-update dates, routing rules, system prompts, retrieval pipelines, training data, decoding parameters, and open- or closed-source status of the deployed backends could not be independently verified. The findings therefore describe the deployed web interfaces under the tested conditions and dates rather than stable properties of verified underlying models.

### 2.3 Question development and selection

HZ terminology was identified using Medical Subject Headings, including the descriptor *Herpes Zoster* and entry terms such as *Shingles*, *Zona*, and *Zoster*. These terms informed an exploratory Google Trends search. Google Trends groups semantically related search terms into topics (27). We used it to identify public information-seeking themes rather than as a quantitative outcome because search activity can reflect temporal changes in public interest (28–30). The initial pool comprised 112 questions derived from public search themes, publicly available patient-education resources, clinical guidelines, medication-safety alerts, and clinically important scenarios. Candidate questions were organised into eight prespecified dimensions of 14 questions each, as detailed in Supplementary material 2. Two dermatologists independently screened the questions for clinician-assessed relevance to patient and public information needs, evidence-based answerability, safety value, non-duplication, lay-language suitability, and alignment with the prespecified evaluation domains. A third dermatologist resolved disagreements. Exclusion criteria were duplicate or near-duplicate content, clinician-facing technical content, commercial recommendations, location- or cost-specific requests, and individualised self-prescription requests. Two overlapping dimensions on pain, recovery, postherpetic neuralgia, and long-term effects were subsequently combined. The final 46 questions covered seven domains: understanding, causes, and risk factors; symptoms, diagnosis, and differential recognition; transmission and daily-life precautions; complications and urgent-care decisions; antiviral treatment and medication safety; pain relief, skin care, recovery, and postherpetic neuralgia; and vaccination, prevention, and special situations. Patients and members of the public were not involved in question development, screening, or validation; therefore, the final set should be regarded as clinician-selected, lay-oriented questions intended to simulate public queries rather than as a patient-developed or patient-validated question set. Question wording, order, and domain assignment were finalised before chatbot querying and were not changed thereafter. Supplementary material 2 contains the screening log and final standardised question set.

### 2.4 Query strategy and response capture

Each standardised English-language question was submitted once to each interface in a separate new conversation. Questions were entered verbatim, without role prompting, examples, follow-up questions, clarification, prompt refinement, regeneration, or study-specific system instructions. The interfaces were queried sequentially in a fixed order, with all 46 questions completed on one interface before the next. No page-loading failure, quota warning, model-switch notification, refusal, network interruption, or incomplete response occurred. Responses were saved verbatim without substantive editing, shortening, rewriting, or reordering. Interface elements unrelated to response content were removed. Complete outputs are provided in Supplementary material 4.

### 2.5 Readability analysis and text preprocessing

We assessed textual readability using six established formula-based indices: Automated Readability Index (ARI) (31), Flesch Reading Ease Score (FRES) (32, 33), Flesch–Kincaid Grade Level (FKGL) (32), Gunning Fog Index (GFI) (32), Coleman-Liau Index (CL) (34), and Simple Measure of Gobbledygook (SMOG) (35). Higher ARI, CL, FKGL, GFI, and SMOG values indicate greater reading difficulty, whereas higher FRES values indicate easier text.

Readability was calculated in R using the quanteda and quanteda.textstats packages. Analyses used cleaned, structured response text. URLs, complete reference lists, citation-only formatting, editorial labels, and standalone headings were removed. Paragraph and list boundaries were retained. Substantive off-topic or redundant content was also retained because it was visible to users. Non-English segments were excluded from the English-language calculations. FRES values were constrained to the conventional 0–100 range before analysis.

### 2.6 Manual outcomes: accuracy, empathy, and safety

Clinical accuracy was rated on a 5-point ordinal scale used in previous evaluations of generative-AI responses (36). Scores represented predominantly incorrect or misleading information (1), substantial inaccuracies or omissions (2), generally accurate information with notable limitations (3), largely accurate information with minor limitations (4), or guideline-consistent information without important inaccuracies or omissions (5). Empathy was independently rated from 1 (dismissive, judgmental, or emotionally inappropriate) to 5 (highly compassionate, non-stigmatising, and responsive to the user’s concern). Ratings considered the clinical context of each question. Safety was prespecified as a binary outcome: 0 indicated safe and 1 indicated potentially unsafe or harmful. A response could be rated unsafe because of an explicit error or a clinically important omission. Safety checkpoints included urgent triage, antiviral and analgesic safety, high-risk exposure precautions, vaccination eligibility and timing, pregnancy and breastfeeding, transplantation, and immunocompromised states (4).

Accuracy ratings were guided by a prespecified question–evidence mapping framework provided in Supplementary material 3. The framework was developed by the research team and independently reviewed by two dermatologists using relevant clinical guidelines, consensus statements, medication-safety information, and other authoritative clinical sources. Disagreements regarding evidence interpretation, key answer points, or clinically acceptable alternatives were resolved by a third dermatologist. The framework was finalised before chatbot querying and was not modified in response to the generated outputs. Because clinically appropriate answers to some herpes zoster questions may depend on factors such as timing of presentation, immune status, pregnancy, transplantation status, treatment eligibility, or other patient-specific circumstances, responses were not judged against a single fixed “gold-standard” answer. Instead, raters assessed whether each response was consistent with the applicable evidence, appropriately qualified when recommendations were context dependent, and free from clinically important inaccuracies or omissions. Accordingly, the accuracy score represents an expert, evidence-anchored assessment rather than an objective percentage of correct statements.

### 2.7 Information quality and transparency measures

Readability findings were interpreted against the commonly recommended sixth-grade target for patient-facing materials (18). For FRES, 80 was used only as a descriptive reference corresponding to the lower bound of the conventional 80–89 “easy” range, which approximates sixth-grade material (33). It was not treated as a validated clinical cutoff and was not used for hypothesis testing. Information quality, response-level source attribution and disclosure, and overall quality were assessed using DISCERN, EQIP, the JAMA benchmark criteria, and GQS.

DISCERN: Treatment-related information was assessed using the 16-item DISCERN instrument (37). Each item was scored from 1 to 5, yielding a total score of 16–80. Because DISCERN has no official categorical classification, scores were analysed continuously. Previously published tiered interpretations were considered descriptively only (38).

Ensuring Quality Information for Patients (EQIP): Patient-oriented information quality was assessed using the 20-item EQIP instrument, which evaluates content, clarity, structure, and writing style (39). Each item was scored as 0 or 5, yielding a total score of 0–100. Because published EQIP scoring and interpretation methods vary (39, 40), scores were analysed continuously, with higher values indicating better quality.

Global Quality Score (GQS): GQS provided a single 1–5 rating of overall quality, coherence, flow, and usefulness, using the five-point Global Quality Score introduced by Bernard et al. (41). Higher scores indicate better overall quality and greater usefulness. The GQS was treated as an ordinal global rating and was not described as a formally validated instrument.

JAMA benchmark: Response-level source attribution and disclosure were assessed using the JAMA benchmark criteria (42). Four binary items evaluated authorship, attribution, disclosure, and currency, yielding a total score of 0–4. JAMA scores were not interpreted as a comprehensive measure of model transparency.

### 2.8 Raters, training, blinding, and final-score determination

Five dermatologists independently evaluated all 230 responses using an online scoring system, predefined scoring anchors, and the question–evidence mapping framework provided in Supplementary material 3. Before formal assessment, the raters received standardised training and completed a calibration exercise using responses that were excluded from the final analysis. Responses were randomly ordered and de-identified before evaluation. Model names and other explicit interface identifiers were removed; however, complete blinding could not be guaranteed because citation styles, formatting patterns, response structure, or self-identifying language could potentially reveal the originating interface. The evaluation was therefore considered partially blinded. Inter-rater agreement was calculated from the five independent ratings before any consensus resolution. Final response-level scores were determined using prespecified majority rules rather than arithmetic means.

For Safety, a response was classified as potentially unsafe when at least 3 of the 5 independent dermatologists assigned a value of 1, representing an absolute majority of the expert ratings. This threshold was selected to require concordance of more than half of the independent expert panel while preventing a single or minority rating from determining the final response-level classification.

For Accuracy, Empathy, and GQS, the modal integer rating was retained when an absolute majority was achieved. DISCERN, EQIP, and JAMA were resolved at the item level by majority rating and then summed to obtain the total score. When no absolute majority was achieved for a nonbinary rating or item, the raters conducted a consensus review while the interface identity remained concealed and retained a single integer score.

### 2.9 Safety qualitative analysis

Responses classified as potentially unsafe under the prespecified majority rule were reviewed qualitatively at the question–interface level. A response was flagged when an explicit inaccuracy, clinically important omission, nonresponse, unsupported certainty, or ambiguous instruction could plausibly alter patient behaviour, delay urgent assessment, promote inappropriate self-management, or distort vaccination decisions. A flagged response did not imply that every statement in the answer was incorrect. Safety concerns were categorised descriptively according to the possible harm pathway, and the detailed qualitative analysis is provided in Supplementary material 5.

### 2.10 Sample size

The question was the primary paired analytical unit. Submitting 46 questions to five interfaces produced 230 question–interface responses and 46 complete five-interface blocks. The sample size was determined by the prespecified question set and number of interfaces rather than a formal power calculation. A hypothesis-driven calculation was not considered appropriate because the study was exploratory and included multiple outcomes without one prespecified primary effect size. The design balanced clinical coverage, feasibility of five-rater assessment, and paired-analysis requirements. Each question was submitted once per interface; therefore, the study compared first responses under standardised conditions and did not assess within-interface variability or response stability across repeated generations.

### 2.11 Statistical analysis

The question was the paired unit for all between-interface comparisons. This study was designed as a multidimensional exploratory evaluation without a single prespecified primary endpoint; all evaluation domains were prespecified before analysis. Safety was compared across the five matched responses using Cochran’s Q test, and potentially unsafe counts and percentages were reported for each interface. The effect size was calculated as Q/[N(k − 1)], where N was the number of paired questions and k was the number of interfaces.

To assess the robustness of the prespecified majority-based safety classification, a *post hoc* sensitivity analysis was performed using alternative thresholds of ≥2/5 and ≥4/5 unsafe ratings among the five independent dermatologists. For each threshold, interface-specific counts and proportions, the Cochran’s Q statistic, *p* value, and Q/[N(k − 1)] effect size were recalculated using the same analytical procedures as in the main safety analysis.

Accuracy, empathy, DISCERN, EQIP, JAMA, GQS, and the six readability indices were compared using Friedman tests, with Kendall’s W reported as the overall effect size. After a significant Friedman test, all 10 paired interface comparisons were evaluated using Wilcoxon signed-rank tests. Benjamini–Hochberg correction was applied separately to the 10 *p* values for each outcome. Omnibus *p* values were not adjusted across outcomes and were therefore interpreted as exploratory.

Zero differences were excluded from signed-rank calculations, tied absolute differences received average ranks, and asymptotic two-sided *p* values were calculated with continuity correction. Confidence intervals for Kendall’s W, the Cochran’s Q effect size, and paired effects were estimated using 3,000 question-level nonparametric bootstrap resamples with the percentile method.

Safety agreement was assessed using Fleiss’ kappa. Its 95% confidence interval was estimated using 3,000 nonparametric bootstrap resamples of the complete response-level rating data. Agreement for the other manually rated outcomes was assessed using ICC(2,1), corresponding to a two-way random-effects, absolute-agreement, single-measure model (43, 44). ICC (2,1) was selected to estimate the absolute agreement expected for one dermatologist drawn from the broader population of similarly qualified dermatologists, rather than the reliability of the mean rating of this five-member panel. Agreement coefficients were calculated from the independent ratings before consensus resolution.

Descriptive results are reported as mean ± standard deviation and median [first quartile, third quartile]. All 230 responses were complete, and no imputation was performed. Tests were two-sided, with *p* < 0.05 considered statistically significant. Analyses were conducted using R version 4.6.0 (R Foundation for Statistical Computing, Vienna, Austria).

### 2.12 Ethics, privacy, and protocol status

This study analysed responses to standardised, non-personal prompts and did not involve human participants, patient records, private health information, or identifiable data. No patient information was entered into a chatbot. Formal ethics approval was therefore not required under local institutional policy. The evaluated systems were not trained, fine-tuned, or otherwise modified. No formal study protocol was registered or published. The final question set, scoring framework, complete responses, safety-case records, scoring and readability datasets, and analysis code were retained as audit materials and are provided in the Supplementary materials where indicated.

## 3 Results

All five interfaces generated complete responses to the 46 questions, yielding 230 question–interface responses. No response was excluded, and no data were missing.

### 3.1 Inter-rater agreement and consensus resolution

Five dermatologists independently evaluated all 230 responses. Fleiss’ kappa for safety was 0.682 (95% CI, 0.636–0.728; *p* < 0.001). ICC (2,1) estimates for the other manually rated outcomes ranged from 0.832 for GQS to 0.889 for DISCERN (all *p* < 0.001). Detailed inter-rater agreement estimates are provided in Supplementary Table S2.

### 3.2 Safety, accuracy, and empathy

Under the study conditions, 66 of 230 question–interface responses (28.7%) were classified as potentially unsafe using the prespecified ≥3/5 majority threshold. The observed proportions ranged from 23.9 to 32.6% across interfaces, with no evidence of a difference between interfaces (Cochran’s Q = 1.661, df = 4, *p* = 0.798; effect size = 0.009, 95% CI, 0.003–0.084; Table 1; Figure 2). Sensitivity analyses using alternative thresholds of ≥2/5 and ≥4/5 unsafe ratings yielded the same comparative conclusion, with no significant between-interface differences at either threshold (Cochran’s Q = 2.062, *p* = 0.724 and Q = 2.892, *p* = 0.576, respectively; Supplementary Table S3).

**Table 1:** Safety, accuracy, and empathy across the five LLM chatbot interfaces.

Metric	ChatGPT-5.5 Instant	DeepSeek-V4-Pro	Doubao-Seed-2.0-Pro	Gemini 3.5 Flash	Qwen3.7-Plus	*P*	Effect size (95% CI)
Potentially unsafe responses, *n* (%)	11 (23.9)	12 (26.1)	15 (32.6)	14 (30.4)	14 (30.4)	0.798	0.009 (0.003–0.084)
Accuracy median [Q1, Q3]	4.00 [4.00, 4.00]	4.00 [4.00, 4.00]	4.00 [4.00, 4.00]	4.00 [4.00, 4.00]	4.00 [4.00, 4.00]	<0.001	0.127 (0.068–0.222)
Empathy median [Q1, Q3]	4.00 [4.00, 4.00]	3.00 [3.00, 4.00]	3.00 [3.00, 4.00]	3.00 [3.00, 4.00]	3.00 [3.00, 4.00]	<0.001	0.211 (0.124–0.342)

Safety was coded as 0 = safe and 1 = potentially unsafe; the prespecified primary classification required an absolute majority, with ≥3 of 5 raters assigning a value of 1. Cochran’s Q test compared safety, with Q/[N(k − 1)] reported as the effect size. Friedman tests compared accuracy and empathy, with Kendall’s W as the effect size. Pairwise *p* values were adjusted using the Benjamini–Hochberg procedure.

**Figure 2:**
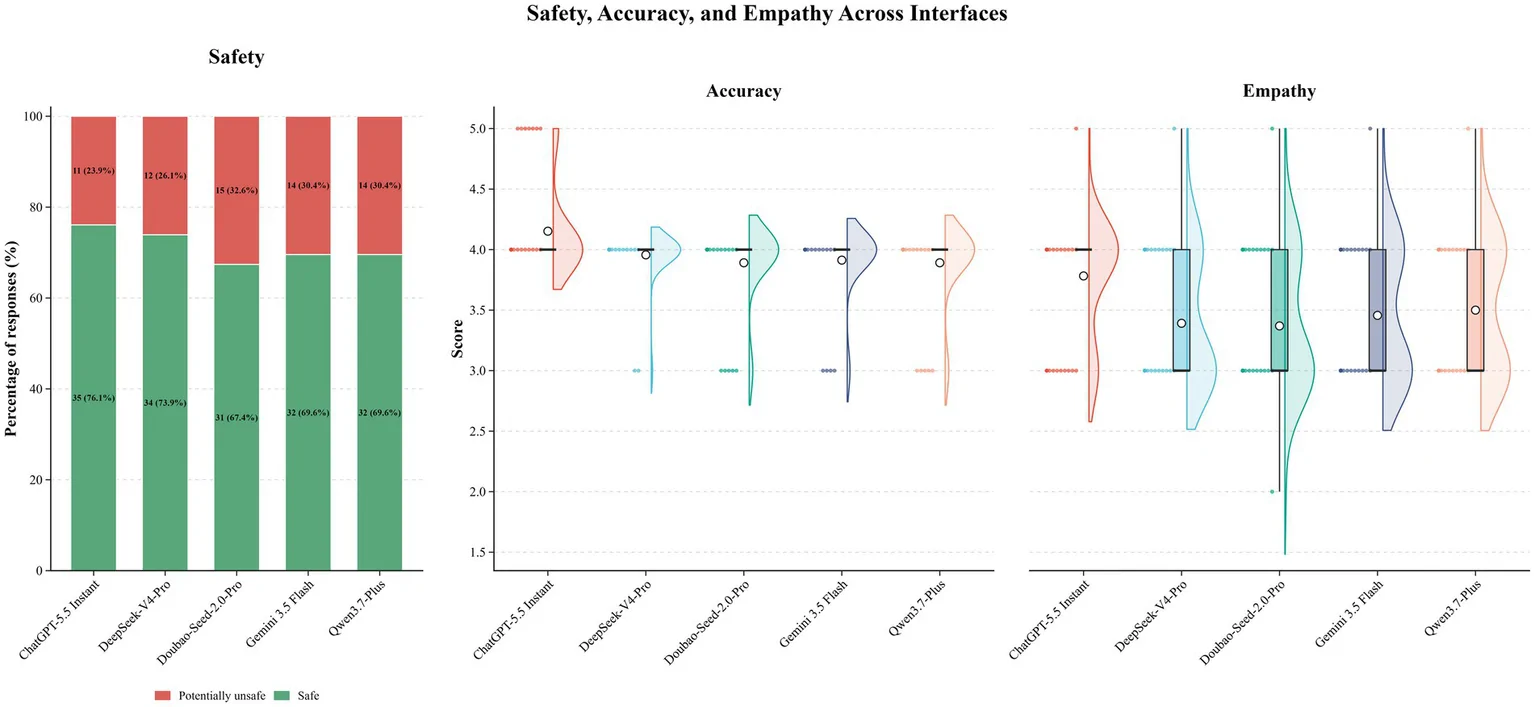
Distributions of safety, accuracy, and empathy across interfaces. The safety panel shows safe and potentially unsafe responses for each interface (*n* = 46 paired questions per interface). Accuracy and empathy panels show question-level observations, box plots, half-violin densities, and means (white circles). Overall comparisons used Cochran’s Q test for safety and Friedman tests for accuracy and empathy. Benjamini–Hochberg correction was applied to *post hoc* paired Wilcoxon signed-rank tests.

Accuracy ratings differed between interfaces (*p* < 0.001; Kendall’s *W* = 0.127, 95% CI, 0.068–0.222), although all interfaces had a median score of 4.00. ChatGPT-5.5 Instant had the highest mean score (4.15 ± 0.36) and higher paired ratings than the other interfaces (adjusted *p* = 0.016–0.044). The overlapping distributions and small effect size indicated limited absolute separation.

Empathy ratings also differed between interfaces (*p* < 0.001; Kendall’s *W* = 0.211, 95% CI, 0.124–0.342). ChatGPT-5.5 Instant had the highest mean score (3.78 ± 0.47) and higher paired ratings than all comparators (adjusted *p* ≤ 0.008). Distributions nevertheless overlapped substantially. Complete *post hoc* pairwise comparisons for accuracy and empathy are provided in Supplementary Table S4.

Safety concerns most often involved household exposure precautions; urgent assessment for ophthalmic, otologic, disseminated, or neurological presentations; antiviral and postherpetic-neuralgia claims; medication dosing and self-management; and vaccination during pregnancy, breastfeeding, transplantation, or immunocompromise.

### 3.3 Information quality and response-level source attribution

DISCERN, EQIP, JAMA, and GQS scores differed between interfaces, although effect sizes were small (Table 2; Figure 3). DISCERN showed a small overall effect (*p* < 0.001; Kendall’s *W* = 0.118, 95% CI, 0.057–0.231), driven mainly by comparisons involving Gemini 3.5 Flash. The EQIP effect was smaller (*p* = 0.035; Kendall’s *W* = 0.056, 95% CI, 0.026–0.151). JAMA had the largest effect among these measures (*p* < 0.001; Kendall’s *W* = 0.225, 95% CI, 0.129–0.369), and Qwen3.7-Plus scored higher than the other interfaces after correction. Absolute JAMA scores were nevertheless low. The highest mean was 0.91 ± 0.78 of 4, indicating limited response-level authorship, attribution, disclosure, and currency information. GQS also differed (*p* < 0.001; Kendall’s *W* = 0.110, 95% CI, 0.058–0.201), but every interface had a median of 4.00. Complete *post hoc* pairwise comparisons for DISCERN, EQIP, JAMA, and GQS are provided in Supplementary Table S5.

**Table 2:** Information quality, response-level source attribution and disclosure, and overall quality across the five LLM chatbot interfaces.

Metric	ChatGPT-5.5 Instant	DeepSeek-V4-Pro	Doubao-Seed-2.0-Pro	Gemini 3.5 Flash	Qwen3.7-Plus	*P*	Effect size (95% CI)
DISCERN median [Q1, Q3]	51.00 [45.25, 57.00]	48.00 [45.00, 57.00]	48.50 [42.25, 57.75]	47.00 [43.00, 55.00]	50.00 [46.25, 57.00]	<0.001	0.118 (0.057–0.231)
EQIP median [Q1, Q3]	85.00 [81.25, 90.00]	85.00 [75.00, 85.00]	85.00 [75.00, 85.00]	82.50 [75.00, 90.00]	85.00 [80.00, 85.00]	0.035	0.056 (0.026–0.151)
JAMA median [Q1, Q3]	0.00 [0.00, 1.00]	0.00 [0.00, 1.00]	0.00 [0.00, 1.00]	0.00 [0.00, 0.00]	1.00 [0.00, 1.00]	<0.001	0.225 (0.129–0.369)
GQS median [Q1, Q3]	4.00 [4.00, 4.00]	4.00 [4.00, 4.00]	4.00 [4.00, 4.00]	4.00 [4.00, 4.00]	4.00 [4.00, 4.00]	<0.001	0.110 (0.058–0.201)

Values are median [Q1, Q3] for 46 paired questions per interface. Friedman tests provided overall *p* values; Kendall’s W and bootstrap 95% CIs are reported as effect sizes. Higher scores indicate better performance. JAMA scores were interpreted as indicators of response-level source attribution and disclosure, not comprehensive model transparency.

**Figure 3:**
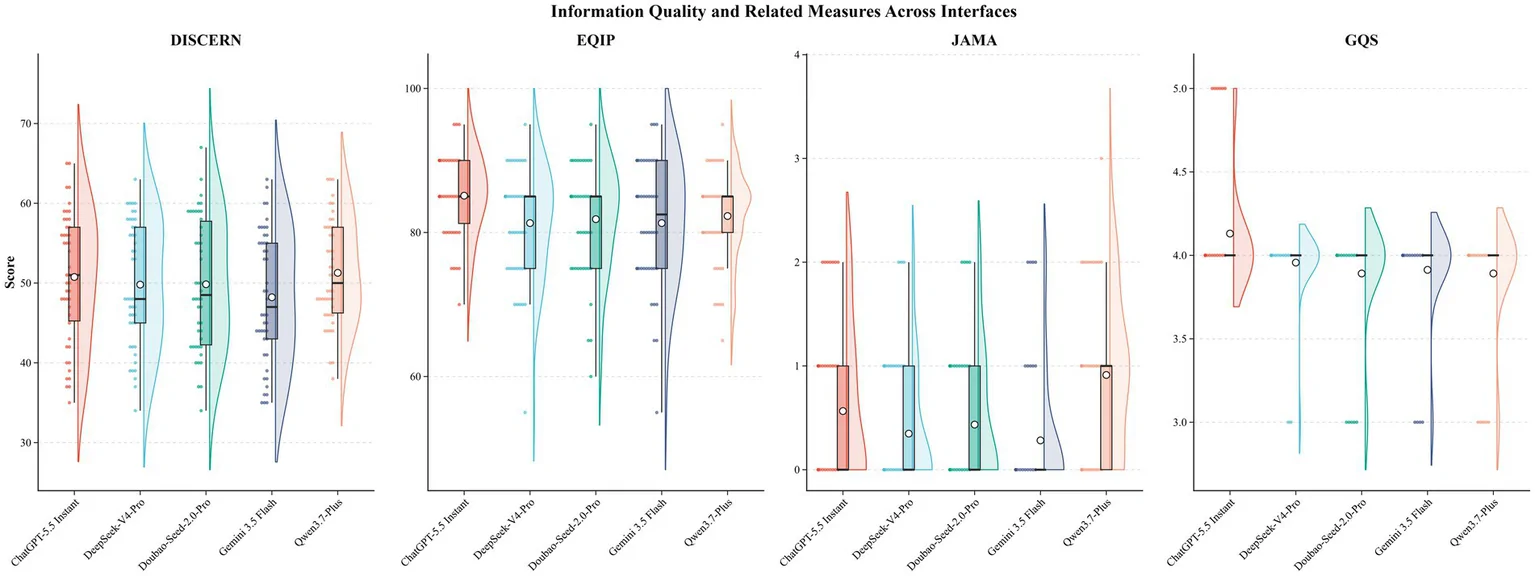
Distributions of information-quality, source-attribution, disclosure, and overall-quality scores across interfaces. Panels show DISCERN, EQIP, JAMA, and GQS values for 46 paired questions per interface. Points are question-level observations; box plots show medians and interquartile ranges; half-violin curves show densities; and white circles show means. Overall comparisons used Friedman tests. Benjamini–Hochberg correction was applied to *post hoc* paired Wilcoxon signed-rank tests.

### 3.4 Readability

All six readability indices differed between interfaces (all *p* < 0.001), with Kendall’s W ranging from 0.293 to 0.339 (Table 3; Figure 4). DeepSeek-V4-Pro generally produced text estimated to be easier to read across the grade-level indices and had the highest mean FRES score (47.33 ± 12.60). Gemini 3.5 Flash generally produced text estimated to be more difficult to read, with the highest ARI, CL, FKGL, GFI, and SMOG values and the lowest FRES score (31.19 ± 16.63). After correction, DeepSeek-V4-Pro had lower ARI, FKGL, GFI, and SMOG values than ChatGPT-5.5 Instant; CL and FRES did not differ. ChatGPT-5.5 Instant and DeepSeek-V4-Pro had higher FRES scores than Doubao-Seed-2.0-Pro, Gemini 3.5 Flash, and Qwen3.7-Plus. Qwen3.7-Plus generally had intermediate values. Despite these relative differences, all interface-level mean grade estimates exceeded the sixth-grade target, and all mean FRES values were below 80. Complete *post hoc* pairwise comparisons for all six readability indices are provided in Supplementary Table S6.

**Table 3:** Readability scores across the five LLM chatbot interfaces.

Metric	ChatGPT-5.5 Instant	DeepSeek-V4-Pro	Doubao-Seed-2.0-Pro	Gemini 3.5 Flash	Qwen3.7-Plus	*P*	Effect size (95% CI)
ARI mean ± SD	12.42 ± 2.66	10.26 ± 2.49	11.57 ± 2.30	14.18 ± 2.99	12.70 ± 2.22	<0.001	0.318 (0.225–0.456)
CL mean ± SD	12.53 ± 1.95	12.24 ± 2.05	14.31 ± 1.93	14.80 ± 2.50	13.41 ± 1.86	<0.001	0.339 (0.251–0.463)
FKGL mean ± SD	11.83 ± 2.28	10.42 ± 2.25	11.77 ± 2.28	13.85 ± 2.98	12.34 ± 1.93	<0.001	0.293 (0.201–0.419)
GFI mean ± SD	13.99 ± 2.63	12.93 ± 2.54	14.39 ± 2.63	16.67 ± 3.32	15.08 ± 2.25	<0.001	0.308 (0.220–0.426)
SMOG mean ± SD	12.84 ± 1.91	12.19 ± 1.71	13.09 ± 1.81	14.93 ± 2.35	13.76 ± 1.62	<0.001	0.304 (0.217–0.430)
FRES mean ± SD	45.48 ± 11.54	47.33 ± 12.60	36.07 ± 13.23	31.19 ± 16.63	40.20 ± 10.81	<0.001	0.321 (0.222–0.446)

Values are mean ± SD for 46 paired questions per interface. Friedman tests provided overall *p* values; Kendall’s W and bootstrap 95% CIs are reported as effect sizes. Higher FRES values indicate easier text; higher ARI, CL, FKGL, GFI, and SMOG values indicate greater reading difficulty.

**Figure 4:**
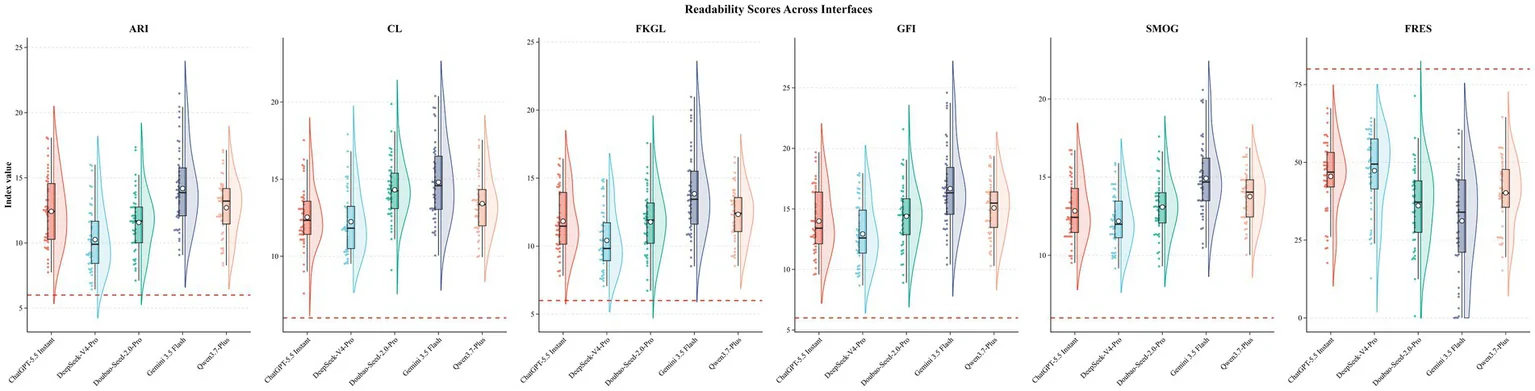
Distributions of readability scores across interfaces. Panels show ARI, CL, FKGL, GFI, SMOG, and FRES values for 46 paired questions per interface. Higher FRES values indicate easier text; higher values for the other indices indicate greater difficulty. Points are question-level observations; box plots show medians and interquartile ranges; half-violin curves show densities; and white circles show means. Red dashed lines mark descriptive reference values of 6 for grade-level indices and 80 for FRES. FRES = 80 is the lower bound of the conventional 80–89 “easy” range and was not used as a validated clinical cutoff or inferential threshold. Overall comparisons used Friedman tests. Benjamini-Hochberg correction was applied to *post hoc* paired Wilcoxon signed-rank tests.

## 4 Discussion

### 4.1 Principal findings

This study evaluated five consumer-facing LLM chatbot interfaces using 46 English-language questions about common and safety-relevant aspects of HZ. The findings revealed a gap between favourable quality ratings and the requirements of dependable patient-facing information. Within this selected benchmark, which intentionally included common as well as safety-critical and clinically important scenarios, 66 of 230 responses (28.7%) were classified as potentially unsafe under the study conditions. This proportion should not be interpreted as an estimate of the prevalence of unsafe responses to HZ-related queries in routine public use. Performance varied by outcome, and no interface consistently performed best across safety, accuracy, empathy, information quality, source attribution and disclosure, overall quality, and readability. Most effect sizes were small to moderate. This outcome-specific pattern is consistent with prior health-advice evaluations, in which heterogeneous interfaces, prompts, questions, instruments, and reporting methods limit direct platform rankings (45).

The clinical relevance of reliable herpes zoster information is particularly important because patients frequently seek online information regarding symptoms, complications, treatment, and vaccination decisions. Inaccurate, incomplete, or difficult-to-understand information may influence health-related decisions, especially among older adults and individuals with higher disease risk.

### 4.2 Information quality and source attribution and disclosure

Information quality and response-level source attribution and disclosure captured distinct aspects of performance. DISCERN, EQIP, and GQS suggested that many responses were useful and well structured. In contrast, low JAMA scores indicated limited authorship, attribution, disclosure, and currency information. A comprehensive answer may therefore remain difficult for users to verify. Response-level attribution and disclosure should not be equated with model transparency, which also concerns training data, system governance, and algorithmic accountability. Other patient-education studies have also found that reliability, readability, understandability, and actionability do not consistently favour the same interface (46, 47). Evaluations should therefore use complementary outcomes rather than a single composite quality score. The relevant question is not which chatbot is universally best, but whether a specific response is accurate, complete, appropriately attributed, and presented at an appropriate reading level for its intended audience.

### 4.3 Accuracy, safety, and clinical outcomes are distinct constructs

Accuracy and safety are related but distinct constructs. Every interface had a median accuracy score of 4.00, yet 66 of 230 responses were classified as potentially unsafe under the study conditions. Clinically plausible information can therefore remain risky when it omits an important safeguard. Safety concerns mainly involved insufficient escalation advice for ophthalmic, otologic, disseminated, or neurologic presentations; incomplete discussion of medication precautions; inappropriate self-management suggestions; and inadequate qualification of vaccination recommendations for pregnancy, breastfeeding, transplantation, and immunocompromised conditions. Previous studies have likewise shown that favourable average accuracy does not eliminate clinically important errors or response variability (48). AI-generated educational content can cover useful information while retaining omissions or inappropriate recommendations that require professional review (47). Accuracy is also not equivalent to clinical effectiveness. A response may receive favourable empathy or quality ratings without improving comprehension, decisions, adherence, or health outcomes. Comparisons of chatbot and physician responses suggest that acceptable communication quality does not remove the need for clinical oversight (36, 49). Our findings indicate plausible informational risks rather than observed patient harm. LLM-generated HZ information may support patient-education workflows, but it should not replace individual clinical assessment.

### 4.4 Readability challenges

Readability remained a limitation for every interface. All interface-level means exceeded recommended reading levels, despite significant between-interface differences. Similar findings have been reported for cardiopulmonary resuscitation, otolaryngology, oral health, diabetes technology, and bariatric surgery (50–55). Relative performance should not be mistaken for adequate readability. DeepSeek-V4-Pro produced less difficult text on several indices, but its average values still exceeded the recommended threshold. No evaluated interface consistently produced language suitable for general patient education. Controlled prompting and post-generation editing may improve readability. Targeted reading-level prompts and AI-assisted simplification have improved the readability of patient materials (56–58). Simplification can nevertheless remove clinically important detail or nuance. Future studies should evaluate clinician-reviewed, source-grounded, and readability-controlled responses rather than default outputs alone.

### 4.5 Practical implications

For clinicians, chatbot-generated HZ information may provide a preliminary draft or educational aid. However, the observed safety limitations and inadequate source attribution do not support unreviewed direct patient communication. Clinical review is needed to verify recommendations, identify missing safeguards, and adapt content to individual circumstances and health-literacy needs.

For patients and the public, chatbots may provide conversational explanations and help users prepare questions for healthcare professionals. Users may nevertheless struggle to judge whether information is complete, current, appropriately attributed, or personally applicable. Professional assessment remains important for high-risk presentations, individual treatment decisions, and vaccination questions.

For developers, improving factual accuracy alone may not ensure safe patient-facing use. Useful priorities include evidence-based retrieval, clearer source and update information, risk-sensitive safeguards, uncertainty communication, and adjustable reading levels. Because greater guideline alignment can increase linguistic complexity (47), safety, completeness, and readability should be considered together.

A layered response may help: a concise explanation and key safety actions can appear first, followed by detailed information and supporting sources.

### 4.6 Strengths and limitations

This study has several strengths. Identical questions and first-response conditions enabled paired comparisons across five public chatbot interfaces. The evaluation integrated safety, accuracy, empathy, information quality, source attribution and disclosure, overall quality, and readability. Expert quantitative ratings were complemented by a qualitative review of potentially unsafe responses.

Several limitations require consideration. The question set covered common and safety-relevant HZ concerns but may not represent the full range of patient needs. Researchers and dermatologists selected the final questions from public search themes, educational resources, guidelines, safety information, and clinical scenarios. Patients and members of the public did not participate. The wording and priorities may therefore differ from those of real users. Only one response was generated for each question–interface pair. The study therefore provides a single-response snapshot rather than an estimate of response stability. It did not examine repeated prompts, regeneration, follow-up interaction, multi-turn conversations, or later system updates. Because interfaces were queried sequentially in a fixed order over 2 days, interface and collection time could not be fully separated.

The evaluation was limited to English-language responses from consumer interfaces during a defined period. Performance may differ by language, location, access conditions, retrieval settings, personalisation, and future updates. Heterogeneous evaluation and reporting methods also limit comparisons across chatbot studies (45).

The evaluated systems were deployed interfaces rather than verified model checkpoints. System prompts, retrieval pipelines, generation parameters, routing, and backend updates were not fully accessible. The results therefore describe specific user-facing experiences rather than fixed underlying models. DISCERN, EQIP, JAMA, and GQS were not developed specifically for conversational generative AI and required adaptation. The modified EQIP scoring method should not be treated as equivalent to the original validated method. JAMA scores represent only response-level source attribution and disclosure.

Expert ratings remained subjective despite training, calibration, prespecified criteria, and majority rules. Complete masking was not possible because structure, citation style, or formatting could reveal an interface. Readability formulas measure surface linguistic complexity, not comprehension, cultural suitability, or recognition of urgent information.

Finally, the study evaluated generated text rather than clinical use. It did not assess patient comprehension, confidence, care seeking, treatment decisions, vaccination uptake, adverse events, or clinical outcomes. Potentially unsafe classifications therefore indicate plausible informational risk, not documented harm. Future studies should include patient involvement, repeated and multi-turn testing, multilingual evaluation, comprehension outcomes, and comparisons with clinician-reviewed or guideline-grounded systems.

## 5 Conclusion

In this paired evaluation of five consumer-facing LLM chatbot interfaces, responses to clinician-selected, lay-oriented HZ questions generally received favourable accuracy and quality ratings. Clinically relevant safety limitations, weak response-level source attribution and disclosure, and substantial readability barriers nevertheless remained.

Differences were outcome-specific, and no interface consistently performed best across safety, accuracy, empathy, information quality, source attribution and disclosure, and readability. Fluent and informative output should not be treated as equivalent to verified, clinically appropriate patient information. LLM-generated HZ information could be explored as a supplementary source or as material for clinician-reviewed content development, but its educational usefulness and effects on patient comprehension require direct evaluation. It should not replace individualised professional assessment or professionally reviewed patient information.

## Code availability

The R scripts used for readability calculation, statistical analysis, and figure generation are provided in the Supplementary materials. No custom model code, model weights, application programming interfaces, or model-generation parameters were used or available because all systems were accessed manually through official provider-hosted consumer web interfaces.

## Guarantor

HLi is the guarantor of this work, had full access to the study materials, and takes responsibility for the integrity of the data and accuracy of the analysis.

## Data Availability

The original contributions presented in the study are included in the article/Supplementary material, further inquiries can be directed to the corresponding author.
