# Supplementary material for: A cross-sectional evaluation of large language model chatbot interfaces for patient-facing herpes zoster information: safety, information quality, and readability
Source: Front Public Health. 2026 Sep 16;14:1955701. doi: 10.3389/fpubh.2026.1955701 (PMC13623941; doi:10.3389/fpubh.2026.1955701)
Supplement: Supplementary file 1 [file Supplementary_file_1.docx]

**Supplementary Tables**

**Supplementary Table S1. Web-interface access and displayed labels**

All five systems were queried manually on 2–3 June 2026 in Guangzhou, China (UTC+8) through official provider-hosted consumer websites using free accounts. No application programming interface (API) was used.

| **Provider / web interface** | **Displayed web-interface label** | **Access method** | **User-controlled query conditions** | **Unobservable deployment details** |
| --- | --- | --- | --- | --- |
| OpenAI / ChatGPT | ChatGPT-5.5 Instant | Official provider-hosted consumer website; free account; manual entry; no API | One question per new conversation; displayed label manually selected; provider defaults retained; no follow-up prompting or regeneration | Backend checkpoint, routing, system prompt, retrieval behaviour, and decoding parameters were not exposed; the displayed label documents the tested web interface only |
| DeepSeek | DeepSeek-V4-Pro | Official provider-hosted consumer website; free account; manual entry; no API | One question per new conversation; displayed label manually selected; provider defaults retained; no follow-up prompting or regeneration | Backend checkpoint, routing, system prompt, retrieval behaviour, and decoding parameters were not exposed; the displayed label documents the tested web interface only |
| ByteDance / Doubao | Doubao-Seed-2.0-Pro | Official provider-hosted consumer website; free account; manual entry; no API | One question per new conversation; displayed label manually selected; provider defaults retained; no follow-up prompting or regeneration | Backend checkpoint, routing, system prompt, retrieval behaviour, and decoding parameters were not exposed; the displayed label documents the tested web interface only |
| Google / Gemini | Gemini 3.5 Flash | Official provider-hosted consumer website; free account; manual entry; no API | One question per new conversation; displayed label manually selected; provider defaults retained; no follow-up prompting or regeneration | Backend checkpoint, routing, system prompt, retrieval behaviour, and decoding parameters were not exposed; the displayed label documents the tested web interface only |
| Alibaba / Qwen | Qwen3.7-Plus | Official provider-hosted consumer website; free account; manual entry; no API | One question per new conversation; displayed label manually selected; provider defaults retained; no follow-up prompting or regeneration | Backend checkpoint, routing, system prompt, retrieval behaviour, and decoding parameters were not exposed; the displayed label documents the tested web interface only |

Official consumer web interfaces: ChatGPT, https://chatgpt.com/; DeepSeek, https://chat.deepseek.com/; Doubao, https://www.doubao.com/chat/; Gemini, https://gemini.google.com/; and Qwen, https://chat.qwen.ai/. All interfaces were accessed manually using free consumer accounts during data collection on 2–3 June 2026; no API or third-party aggregator was used.

**Supplementary Table S2. Inter-rater agreement for human-rated outcomes**

| **Outcome** | **Agreement index** | **Estimate** | **95% CI** | **P value** | **Complete responses** | **No. of raters** |
| --- | --- | --- | --- | --- | --- | --- |
| Safety | Fleiss' kappa | 0.682 | 0.636-0.728 | <0.001 | 230 | 5 |
| Accuracy | ICC(2,1) | 0.866 | 0.840-0.889 | <0.001 | 230 | 5 |
| Empathy | ICC(2,1) | 0.867 | 0.841-0.890 | <0.001 | 230 | 5 |
| DISCERN | ICC(2,1) | 0.889 | 0.728-0.943 | <0.001 | 230 | 5 |
| EQIP | ICC(2,1) | 0.856 | 0.780-0.901 | <0.001 | 230 | 5 |
| JAMA | ICC(2,1) | 0.864 | 0.834-0.891 | <0.001 | 230 | 5 |
| GQS | ICC(2,1) | 0.832 | 0.801-0.861 | <0.001 | 230 | 5 |

***Note:*** *Safety agreement was assessed using Fleiss' kappa; its 95% CI was estimated using 3,000 nonparametric bootstrap resamples of the complete response-level rating data. Accuracy, Empathy, DISCERN, EQIP, JAMA, and GQS were assessed using ICC(2,1), corresponding to a two-way random-effects, absolute-agreement, single-measure model; the reported ICC estimates are shown with their corresponding 95% CIs.*

**Supplementary Table S3. Sensitivity analyses of safety classification**

| **Safety threshold** | **ChatGPT-5.5 Instant** | **DeepSeek-V4-Pro** | **Doubao-Seed-2.0-Pro** | **Gemini 3.5 Flash** | **Qwen3.7-Plus** | **Overall potentially unsafe** | **Cochran Q** | **P value** | **Effect size (95% CI)** |
| --- | --- | --- | --- | --- | --- | --- | --- | --- | --- |
| >=2/5 raters | 11 (23.9%) | 12 (26.1%) | 15 (32.6%) | 15 (32.6%) | 14 (30.4%) | 67/230 (29.1%) | 2.062 | 0.724 | 0.011 (0.004-0.088) |
| >=3/5 raters (primary) | 11 (23.9%) | 12 (26.1%) | 15 (32.6%) | 14 (30.4%) | 14 (30.4%) | 66/230 (28.7%) | 1.661 | 0.798 | 0.009 (0.003-0.084) |
| >=4/5 raters | 9 (19.6%) | 10 (21.7%) | 14 (30.4%) | 9 (19.6%) | 12 (26.1%) | 54/230 (23.5%) | 2.892 | 0.576 | 0.016 (0.005-0.095) |

***Note:*** *Values for each interface and overall are n (%). The prespecified primary safety classification required at least 3 of 5 raters to classify a response as potentially unsafe. Alternative thresholds of at least 2 of 5 and at least 4 of 5 raters were examined as post hoc sensitivity analyses. All analyses used 46 paired questions across five interfaces. The effect size is Q/[N(k-1)]; 95% CIs were obtained by question-level nonparametric bootstrap resampling.*

**Supplementary Table S4. Pairwise comparisons for accuracy and empathy**

| **Outcome** | **Comparison** | **N pairs** | **Mean difference (95% CI)** | **Median difference (95% CI)** | **Rank-biserial correlation (95% CI)** | **Adjusted P** |
| --- | --- | --- | --- | --- | --- | --- |
| Accuracy | ChatGPT-5.5 Instant vs DeepSeek-V4-Pro | 46 | 0.20 (0.07-0.35) | 0.00 (0.00-0.00) | 1.000 (1.000-1.000) | 0.044 |
| Accuracy | ChatGPT-5.5 Instant vs Doubao-Seed-2.0-Pro | 46 | 0.26 (0.13-0.41) | 0.00 (0.00-0.00) | 1.000 (1.000-1.000) | 0.016 |
| Accuracy | ChatGPT-5.5 Instant vs Gemini 3.5 Flash | 46 | 0.24 (0.11-0.39) | 0.00 (0.00-0.00) | 1.000 (1.000-1.000) | 0.023 |
| Accuracy | ChatGPT-5.5 Instant vs Qwen3.7-Plus | 46 | 0.26 (0.11-0.43) | 0.00 (0.00-0.00) | 1.000 (1.000-1.000) | 0.023 |
| Accuracy | DeepSeek-V4-Pro vs Doubao-Seed-2.0-Pro | 46 | 0.07 (-0.02-0.17) | 0.00 (0.00-0.00) | 0.600 (-0.333-1.000) | 0.388 |
| Accuracy | DeepSeek-V4-Pro vs Gemini 3.5 Flash | 46 | 0.04 (0.00-0.11) | 0.00 (0.00-0.00) | 1.000 (0.000-1.000) | 0.494 |
| Accuracy | DeepSeek-V4-Pro vs Qwen3.7-Plus | 46 | 0.07 (0.00-0.13) | 0.00 (0.00-0.00) | 1.000 (0.000-1.000) | 0.298 |
| Accuracy | Doubao-Seed-2.0-Pro vs Gemini 3.5 Flash | 46 | -0.02 (-0.11-0.07) | 0.00 (0.00-0.00) | -0.200 (-1.000-1.000) | 0.851 |
| Accuracy | Doubao-Seed-2.0-Pro vs Qwen3.7-Plus | 46 | 0.00 (-0.11-0.11) | 0.00 (0.00-0.00) | 0.000 (-1.000-1.000) | 1.000 |
| Accuracy | Gemini 3.5 Flash vs Qwen3.7-Plus | 46 | 0.02 (-0.07-0.13) | 0.00 (0.00-0.00) | 0.200 (-1.000-1.000) | 0.851 |
| Empathy | ChatGPT-5.5 Instant vs DeepSeek-V4-Pro | 46 | 0.39 (0.26-0.54) | 0.00 (0.00-1.00) | 1.000 (1.000-1.000) | <0.001 |
| Empathy | ChatGPT-5.5 Instant vs Doubao-Seed-2.0-Pro | 46 | 0.41 (0.26-0.57) | 0.00 (0.00-1.00) | 1.000 (1.000-1.000) | <0.001 |
| Empathy | ChatGPT-5.5 Instant vs Gemini 3.5 Flash | 46 | 0.33 (0.20-0.46) | 0.00 (0.00-0.00) | 1.000 (1.000-1.000) | <0.001 |
| Empathy | ChatGPT-5.5 Instant vs Qwen3.7-Plus | 46 | 0.28 (0.11-0.43) | 0.00 (0.00-0.50) | 0.684 (0.333-1.000) | 0.008 |
| Empathy | DeepSeek-V4-Pro vs Doubao-Seed-2.0-Pro | 46 | 0.02 (-0.11-0.15) | 0.00 (0.00-0.00) | 0.111 (-0.600-0.778) | 0.790 |
| Empathy | DeepSeek-V4-Pro vs Gemini 3.5 Flash | 46 | -0.07 (-0.17-0.02) | 0.00 (0.00-0.00) | -0.600 (-1.000-0.333) | 0.291 |
| Empathy | DeepSeek-V4-Pro vs Qwen3.7-Plus | 46 | -0.11 (-0.24-0.02) | 0.00 (0.00-0.00) | -0.455 (-1.000-0.143) | 0.207 |
| Empathy | Doubao-Seed-2.0-Pro vs Gemini 3.5 Flash | 46 | -0.09 (-0.17--0.02) | 0.00 (0.00-0.00) | -1.000 (-1.000--1.000) | 0.120 |
| Empathy | Doubao-Seed-2.0-Pro vs Qwen3.7-Plus | 46 | -0.13 (-0.26-0.00) | 0.00 (0.00-0.00) | -0.600 (-1.000-0.000) | 0.120 |
| Empathy | Gemini 3.5 Flash vs Qwen3.7-Plus | 46 | -0.04 (-0.15-0.09) | 0.00 (0.00-0.00) | -0.250 (-1.000-0.500) | 0.589 |

***Note:*** *Pairwise comparisons used paired Wilcoxon signed-rank tests. Benjamini-Hochberg correction was applied separately to the 10 comparisons for each outcome. Differences are calculated as the first-listed interface minus the second-listed interface; positive rank-biserial correlations therefore favour the first-listed interface. Difference estimates and rank-biserial correlations are reported with 3,000-resample paired bootstrap 95% CIs.*

**Supplementary Table S5. Pairwise comparisons for information quality and related measures**

| **Outcome** | **Comparison** | **N pairs** | **Mean difference (95% CI)** | **Median difference (95% CI)** | **Rank-biserial correlation (95% CI)** | **Adjusted P** |
| --- | --- | --- | --- | --- | --- | --- |
| DISCERN | ChatGPT-5.5 Instant vs DeepSeek-V4-Pro | 46 | 0.96 (-0.28-2.22) | 1.00 (0.00-2.00) | 0.293 (-0.059-0.626) | 0.166 |
| DISCERN | ChatGPT-5.5 Instant vs Doubao-Seed-2.0-Pro | 46 | 0.91 (-0.35-2.24) | 0.00 (-2.00-2.50) | 0.204 (-0.167-0.509) | 0.319 |
| DISCERN | ChatGPT-5.5 Instant vs Gemini 3.5 Flash | 46 | 2.54 (1.22-4.02) | 2.00 (1.00-3.50) | 0.577 (0.280-0.820) | 0.005 |
| DISCERN | ChatGPT-5.5 Instant vs Qwen3.7-Plus | 46 | -0.52 (-2.02-1.07) | 0.00 (-2.00-1.00) | -0.163 (-0.512-0.200) | 0.411 |
| DISCERN | DeepSeek-V4-Pro vs Doubao-Seed-2.0-Pro | 46 | -0.04 (-1.39-1.37) | 0.00 (-2.50-1.50) | -0.016 (-0.389-0.337) | 0.933 |
| DISCERN | DeepSeek-V4-Pro vs Gemini 3.5 Flash | 46 | 1.59 (0.33-2.85) | 1.50 (0.50-3.00) | 0.396 (0.081-0.683) | 0.078 |
| DISCERN | DeepSeek-V4-Pro vs Qwen3.7-Plus | 46 | -1.48 (-2.96-0.04) | -1.00 (-2.00-0.00) | -0.388 (-0.707--0.033) | 0.096 |
| DISCERN | Doubao-Seed-2.0-Pro vs Gemini 3.5 Flash | 46 | 1.63 (0.11-3.20) | 2.00 (0.00-4.00) | 0.342 (0.010-0.656) | 0.096 |
| DISCERN | Doubao-Seed-2.0-Pro vs Qwen3.7-Plus | 46 | -1.43 (-2.91--0.02) | -0.50 (-3.00-1.00) | -0.302 (-0.601-0.047) | 0.154 |
| DISCERN | Gemini 3.5 Flash vs Qwen3.7-Plus | 46 | -3.07 (-4.54--1.57) | -3.00 (-4.00--2.00) | -0.645 (-0.884--0.371) | 0.002 |
| EQIP | ChatGPT-5.5 Instant vs DeepSeek-V4-Pro | 46 | 3.80 (1.63-5.98) | 5.00 (0.00-5.00) | 0.613 (0.289-0.880) | 0.013 |
| EQIP | ChatGPT-5.5 Instant vs Doubao-Seed-2.0-Pro | 46 | 3.26 (1.30-5.43) | 5.00 (0.00-5.00) | 0.546 (0.227-0.806) | 0.022 |
| EQIP | ChatGPT-5.5 Instant vs Gemini 3.5 Flash | 46 | 3.80 (1.09-6.63) | 2.50 (0.00-5.00) | 0.476 (0.125-0.767) | 0.043 |
| EQIP | ChatGPT-5.5 Instant vs Qwen3.7-Plus | 46 | 2.83 (0.33-5.22) | 2.50 (0.00-5.00) | 0.406 (0.056-0.726) | 0.082 |
| EQIP | DeepSeek-V4-Pro vs Doubao-Seed-2.0-Pro | 46 | -0.54 (-2.72-1.63) | 0.00 (0.00-0.00) | -0.076 (-0.479-0.348) | 0.916 |
| EQIP | DeepSeek-V4-Pro vs Gemini 3.5 Flash | 46 | 0.00 (-2.61-2.61) | 0.00 (-0.06-0.00) | -0.014 (-0.406-0.376) | 0.953 |
| EQIP | DeepSeek-V4-Pro vs Qwen3.7-Plus | 46 | -0.98 (-3.59-1.52) | 0.00 (-5.00-5.00) | -0.108 (-0.460-0.246) | 0.916 |
| EQIP | Doubao-Seed-2.0-Pro vs Gemini 3.5 Flash | 46 | 0.54 (-1.74-3.04) | 0.00 (-5.00-5.00) | 0.057 (-0.304-0.418) | 0.916 |
| EQIP | Doubao-Seed-2.0-Pro vs Qwen3.7-Plus | 46 | -0.43 (-3.04-2.17) | 0.00 (-5.00-5.00) | -0.042 (-0.374-0.323) | 0.916 |
| EQIP | Gemini 3.5 Flash vs Qwen3.7-Plus | 46 | -0.98 (-3.48-1.63) | 0.00 (-5.00-0.00) | -0.165 (-0.521-0.210) | 0.800 |
| JAMA | ChatGPT-5.5 Instant vs DeepSeek-V4-Pro | 46 | 0.22 (0.02-0.39) | 0.00 (0.00-0.00) | 0.500 (0.051-0.895) | 0.058 |
| JAMA | ChatGPT-5.5 Instant vs Doubao-Seed-2.0-Pro | 46 | 0.13 (-0.07-0.35) | 0.00 (0.00-0.00) | 0.276 (-0.187-0.691) | 0.304 |
| JAMA | ChatGPT-5.5 Instant vs Gemini 3.5 Flash | 46 | 0.28 (0.11-0.46) | 0.00 (0.00-0.00) | 0.765 (0.400-1.000) | 0.008 |
| JAMA | ChatGPT-5.5 Instant vs Qwen3.7-Plus | 46 | -0.35 (-0.57--0.13) | 0.00 (-1.00-0.00) | -0.612 (-0.923--0.207) | 0.011 |
| JAMA | DeepSeek-V4-Pro vs Doubao-Seed-2.0-Pro | 46 | -0.09 (-0.26-0.09) | 0.00 (0.00-0.00) | -0.250 (-0.714-0.250) | 0.351 |
| JAMA | DeepSeek-V4-Pro vs Gemini 3.5 Flash | 46 | 0.07 (-0.07-0.20) | 0.00 (0.00-0.00) | 0.333 (-0.333-1.000) | 0.351 |
| JAMA | DeepSeek-V4-Pro vs Qwen3.7-Plus | 46 | -0.57 (-0.78--0.35) | -1.00 (-1.00-0.00) | -0.768 (-0.946--0.529) | <0.001 |
| JAMA | Doubao-Seed-2.0-Pro vs Gemini 3.5 Flash | 46 | 0.15 (0.00-0.30) | 0.00 (0.00-0.00) | 0.467 (0.000-0.867) | 0.108 |
| JAMA | Doubao-Seed-2.0-Pro vs Qwen3.7-Plus | 46 | -0.48 (-0.67--0.26) | 0.00 (-1.00-0.00) | -0.850 (-1.000--0.600) | <0.001 |
| JAMA | Gemini 3.5 Flash vs Qwen3.7-Plus | 46 | -0.63 (-0.83--0.43) | -1.00 (-1.00-0.00) | -0.936 (-1.000--0.778) | <0.001 |
| GQS | ChatGPT-5.5 Instant vs DeepSeek-V4-Pro | 46 | 0.17 (0.04-0.33) | 0.00 (0.00-0.00) | 1.000 (1.000-1.000) | 0.076 |
| GQS | ChatGPT-5.5 Instant vs Doubao-Seed-2.0-Pro | 46 | 0.24 (0.11-0.39) | 0.00 (0.00-0.00) | 1.000 (1.000-1.000) | 0.027 |
| GQS | ChatGPT-5.5 Instant vs Gemini 3.5 Flash | 46 | 0.22 (0.09-0.37) | 0.00 (0.00-0.00) | 1.000 (1.000-1.000) | 0.038 |
| GQS | ChatGPT-5.5 Instant vs Qwen3.7-Plus | 46 | 0.24 (0.09-0.41) | 0.00 (0.00-0.00) | 1.000 (1.000-1.000) | 0.038 |
| GQS | DeepSeek-V4-Pro vs Doubao-Seed-2.0-Pro | 46 | 0.07 (-0.02-0.17) | 0.00 (0.00-0.00) | 0.600 (-0.333-1.000) | 0.388 |
| GQS | DeepSeek-V4-Pro vs Gemini 3.5 Flash | 46 | 0.04 (0.00-0.11) | 0.00 (0.00-0.00) | 1.000 (0.000-1.000) | 0.494 |
| GQS | DeepSeek-V4-Pro vs Qwen3.7-Plus | 46 | 0.07 (0.00-0.15) | 0.00 (0.00-0.00) | 1.000 (0.000-1.000) | 0.298 |
| GQS | Doubao-Seed-2.0-Pro vs Gemini 3.5 Flash | 46 | -0.02 (-0.13-0.07) | 0.00 (0.00-0.00) | -0.200 (-1.000-1.000) | 0.851 |
| GQS | Doubao-Seed-2.0-Pro vs Qwen3.7-Plus | 46 | 0.00 (-0.11-0.11) | 0.00 (0.00-0.00) | 0.000 (-1.000-1.000) | 1.000 |
| GQS | Gemini 3.5 Flash vs Qwen3.7-Plus | 46 | 0.02 (-0.07-0.11) | 0.00 (0.00-0.00) | 0.200 (-1.000-1.000) | 0.851 |

***Note:*** *Pairwise comparisons used paired Wilcoxon signed-rank tests. Benjamini-Hochberg correction was applied separately to the 10 comparisons for each outcome. Differences are calculated as the first-listed interface minus the second-listed interface; positive rank-biserial correlations therefore favour the first-listed interface. Difference estimates and rank-biserial correlations are reported with 3,000-resample paired bootstrap 95% CIs. JAMA values should be interpreted as response-level source attribution and disclosure rather than comprehensive model transparency.*

**Supplementary Table S6. Pairwise comparisons for readability outcomes**

| **Outcome** | **Comparison** | **N pairs** | **Mean difference (95% CI)** | **Median difference (95% CI)** | **Rank-biserial correlation (95% CI)** | **Adjusted P** |
| --- | --- | --- | --- | --- | --- | --- |
| ARI | ChatGPT-5.5 Instant vs DeepSeek-V4-Pro | 46 | 2.16 (1.39-2.94) | 2.08 (1.28-2.48) | 0.758 (0.526-0.915) | <0.001 |
| ARI | ChatGPT-5.5 Instant vs Doubao-Seed-2.0-Pro | 46 | 0.85 (0.02-1.69) | 0.73 (-0.22-2.03) | 0.354 (0.042-0.647) | 0.041 |
| ARI | ChatGPT-5.5 Instant vs Gemini 3.5 Flash | 46 | -1.76 (-2.75--0.81) | -1.61 (-2.43--0.70) | -0.541 (-0.789--0.243) | 0.003 |
| ARI | ChatGPT-5.5 Instant vs Qwen3.7-Plus | 46 | -0.28 (-1.12-0.66) | -0.34 (-1.51-0.30) | -0.186 (-0.504-0.134) | 0.275 |
| ARI | DeepSeek-V4-Pro vs Doubao-Seed-2.0-Pro | 46 | -1.31 (-2.17--0.45) | -1.61 (-2.64--0.66) | -0.484 (-0.743--0.195) | 0.006 |
| ARI | DeepSeek-V4-Pro vs Gemini 3.5 Flash | 46 | -3.92 (-4.77--3.08) | -3.85 (-4.53--3.27) | -0.948 (-0.998--0.858) | <0.001 |
| ARI | DeepSeek-V4-Pro vs Qwen3.7-Plus | 46 | -2.45 (-3.18--1.64) | -2.83 (-3.41--1.87) | -0.802 (-0.950--0.593) | <0.001 |
| ARI | Doubao-Seed-2.0-Pro vs Gemini 3.5 Flash | 46 | -2.61 (-3.64--1.65) | -2.15 (-2.90--1.34) | -0.787 (-0.946--0.578) | <0.001 |
| ARI | Doubao-Seed-2.0-Pro vs Qwen3.7-Plus | 46 | -1.13 (-1.93--0.33) | -1.43 (-2.30-0.42) | -0.419 (-0.680--0.103) | 0.017 |
| ARI | Gemini 3.5 Flash vs Qwen3.7-Plus | 46 | 1.48 (0.56-2.39) | 0.74 (0.35-1.93) | 0.495 (0.204-0.750) | 0.006 |
| CL | ChatGPT-5.5 Instant vs DeepSeek-V4-Pro | 46 | 0.29 (-0.24-0.82) | 0.53 (-0.04-1.10) | 0.191 (-0.134-0.504) | 0.292 |
| CL | ChatGPT-5.5 Instant vs Doubao-Seed-2.0-Pro | 46 | -1.78 (-2.30--1.24) | -2.19 (-2.51--1.49) | -0.808 (-0.961--0.619) | <0.001 |
| CL | ChatGPT-5.5 Instant vs Gemini 3.5 Flash | 46 | -2.27 (-2.97--1.56) | -1.77 (-3.22--0.83) | -0.846 (-0.965--0.669) | <0.001 |
| CL | ChatGPT-5.5 Instant vs Qwen3.7-Plus | 46 | -0.88 (-1.40--0.35) | -0.95 (-1.48--0.10) | -0.517 (-0.771--0.225) | 0.003 |
| CL | DeepSeek-V4-Pro vs Doubao-Seed-2.0-Pro | 46 | -2.07 (-2.58--1.58) | -1.92 (-2.85--1.32) | -0.906 (-0.983--0.778) | <0.001 |
| CL | DeepSeek-V4-Pro vs Gemini 3.5 Flash | 46 | -2.56 (-3.19--1.95) | -2.43 (-2.90--1.88) | -0.930 (-0.989--0.815) | <0.001 |
| CL | DeepSeek-V4-Pro vs Qwen3.7-Plus | 46 | -1.18 (-1.63--0.69) | -1.00 (-2.04--0.76) | -0.685 (-0.874--0.421) | <0.001 |
| CL | Doubao-Seed-2.0-Pro vs Gemini 3.5 Flash | 46 | -0.49 (-1.19-0.19) | -0.06 (-1.05-0.64) | -0.169 (-0.482-0.171) | 0.320 |
| CL | Doubao-Seed-2.0-Pro vs Qwen3.7-Plus | 46 | 0.90 (0.39-1.37) | 1.01 (0.54-1.82) | 0.552 (0.265-0.795) | 0.002 |
| CL | Gemini 3.5 Flash vs Qwen3.7-Plus | 46 | 1.39 (0.71-2.08) | 1.22 (0.06-2.09) | 0.557 (0.278-0.796) | 0.002 |
| FKGL | ChatGPT-5.5 Instant vs DeepSeek-V4-Pro | 46 | 1.41 (0.78-2.08) | 1.38 (0.83-2.12) | 0.641 (0.378-0.856) | <0.001 |
| FKGL | ChatGPT-5.5 Instant vs Doubao-Seed-2.0-Pro | 46 | 0.06 (-0.70-0.80) | -0.21 (-1.28-1.21) | 0.021 (-0.306-0.340) | 0.904 |
| FKGL | ChatGPT-5.5 Instant vs Gemini 3.5 Flash | 46 | -2.01 (-2.94--1.15) | -1.86 (-2.44--0.76) | -0.635 (-0.843--0.373) | <0.001 |
| FKGL | ChatGPT-5.5 Instant vs Qwen3.7-Plus | 46 | -0.50 (-1.21-0.24) | -0.72 (-1.31--0.18) | -0.319 (-0.634-0.003) | 0.075 |
| FKGL | DeepSeek-V4-Pro vs Doubao-Seed-2.0-Pro | 46 | -1.36 (-2.05--0.62) | -1.77 (-2.62--0.06) | -0.537 (-0.778--0.234) | 0.002 |
| FKGL | DeepSeek-V4-Pro vs Gemini 3.5 Flash | 46 | -3.43 (-4.28--2.63) | -3.02 (-4.27--2.57) | -0.946 (-1.000--0.854) | <0.001 |
| FKGL | DeepSeek-V4-Pro vs Qwen3.7-Plus | 46 | -1.92 (-2.52--1.32) | -2.15 (-2.54--1.64) | -0.785 (-0.939--0.576) | <0.001 |
| FKGL | Doubao-Seed-2.0-Pro vs Gemini 3.5 Flash | 46 | -2.07 (-3.02--1.23) | -1.43 (-2.46--0.45) | -0.676 (-0.871--0.436) | <0.001 |
| FKGL | Doubao-Seed-2.0-Pro vs Qwen3.7-Plus | 46 | -0.56 (-1.25-0.11) | -0.81 (-1.53-0.91) | -0.217 (-0.515-0.110) | 0.224 |
| FKGL | Gemini 3.5 Flash vs Qwen3.7-Plus | 46 | 1.51 (0.70-2.41) | 0.92 (0.33-2.54) | 0.537 (0.254-0.780) | 0.002 |
| GFI | ChatGPT-5.5 Instant vs DeepSeek-V4-Pro | 46 | 1.06 (0.34-1.78) | 1.07 (0.42-1.77) | 0.497 (0.201-0.763) | 0.005 |
| GFI | ChatGPT-5.5 Instant vs Doubao-Seed-2.0-Pro | 46 | -0.39 (-1.22-0.42) | 0.06 (-1.45-0.64) | -0.123 (-0.443-0.210) | 0.471 |
| GFI | ChatGPT-5.5 Instant vs Gemini 3.5 Flash | 46 | -2.68 (-3.74--1.65) | -2.47 (-2.93--0.94) | -0.702 (-0.891--0.464) | <0.001 |
| GFI | ChatGPT-5.5 Instant vs Qwen3.7-Plus | 46 | -1.09 (-1.81--0.29) | -1.39 (-2.17--0.65) | -0.476 (-0.743--0.182) | 0.006 |
| GFI | DeepSeek-V4-Pro vs Doubao-Seed-2.0-Pro | 46 | -1.46 (-2.21--0.66) | -1.82 (-2.80--0.19) | -0.536 (-0.793--0.230) | 0.003 |
| GFI | DeepSeek-V4-Pro vs Gemini 3.5 Flash | 46 | -3.74 (-4.65--2.89) | -3.21 (-4.30--2.32) | -0.970 (-1.000--0.904) | <0.001 |
| GFI | DeepSeek-V4-Pro vs Qwen3.7-Plus | 46 | -2.15 (-2.77--1.51) | -2.21 (-2.60--1.79) | -0.827 (-0.967--0.623) | <0.001 |
| GFI | Doubao-Seed-2.0-Pro vs Gemini 3.5 Flash | 46 | -2.29 (-3.39--1.25) | -1.52 (-2.63--0.46) | -0.624 (-0.837--0.369) | <0.001 |
| GFI | Doubao-Seed-2.0-Pro vs Qwen3.7-Plus | 46 | -0.69 (-1.47-0.08) | -0.96 (-1.99-0.55) | -0.267 (-0.561-0.073) | 0.129 |
| GFI | Gemini 3.5 Flash vs Qwen3.7-Plus | 46 | 1.59 (0.62-2.65) | 0.84 (0.36-1.58) | 0.502 (0.201-0.752) | 0.005 |
| SMOG | ChatGPT-5.5 Instant vs DeepSeek-V4-Pro | 46 | 0.66 (0.15-1.17) | 0.67 (0.05-1.39) | 0.436 (0.132-0.703) | 0.014 |
| SMOG | ChatGPT-5.5 Instant vs Doubao-Seed-2.0-Pro | 46 | -0.24 (-0.85-0.37) | -0.19 (-0.95-0.43) | -0.108 (-0.428-0.230) | 0.526 |
| SMOG | ChatGPT-5.5 Instant vs Gemini 3.5 Flash | 46 | -2.08 (-2.83--1.35) | -1.70 (-2.35--0.99) | -0.771 (-0.926--0.561) | <0.001 |
| SMOG | ChatGPT-5.5 Instant vs Qwen3.7-Plus | 46 | -0.92 (-1.43--0.38) | -1.11 (-1.64--0.57) | -0.547 (-0.806--0.243) | 0.003 |
| SMOG | DeepSeek-V4-Pro vs Doubao-Seed-2.0-Pro | 46 | -0.90 (-1.44--0.31) | -1.30 (-1.98-0.07) | -0.464 (-0.736--0.154) | 0.009 |
| SMOG | DeepSeek-V4-Pro vs Gemini 3.5 Flash | 46 | -2.74 (-3.40--2.17) | -2.33 (-3.20--1.89) | -0.983 (-1.000--0.935) | <0.001 |
| SMOG | DeepSeek-V4-Pro vs Qwen3.7-Plus | 46 | -1.58 (-2.04--1.10) | -1.66 (-1.98--1.40) | -0.832 (-0.967--0.634) | <0.001 |
| SMOG | Doubao-Seed-2.0-Pro vs Gemini 3.5 Flash | 46 | -1.84 (-2.66--1.10) | -1.49 (-2.09--0.77) | -0.702 (-0.885--0.463) | <0.001 |
| SMOG | Doubao-Seed-2.0-Pro vs Qwen3.7-Plus | 46 | -0.67 (-1.24--0.11) | -0.94 (-1.67-0.47) | -0.376 (-0.656--0.055) | 0.030 |
| SMOG | Gemini 3.5 Flash vs Qwen3.7-Plus | 46 | 1.16 (0.47-1.94) | 0.58 (0.18-1.11) | 0.503 (0.189-0.770) | 0.007 |
| FRES | ChatGPT-5.5 Instant vs DeepSeek-V4-Pro | 46 | -1.85 (-4.88-1.26) | -4.80 (-5.59--0.75) | -0.236 (-0.549-0.082) | 0.165 |
| FRES | ChatGPT-5.5 Instant vs Doubao-Seed-2.0-Pro | 46 | 9.40 (5.75-12.99) | 9.91 (5.92-14.92) | 0.697 (0.449-0.898) | <0.001 |
| FRES | ChatGPT-5.5 Instant vs Gemini 3.5 Flash | 46 | 14.29 (9.84-18.84) | 13.55 (6.70-19.81) | 0.806 (0.610-0.933) | <0.001 |
| FRES | ChatGPT-5.5 Instant vs Qwen3.7-Plus | 46 | 5.27 (2.33-8.29) | 4.77 (2.90-8.79) | 0.550 (0.264-0.800) | 0.002 |
| FRES | DeepSeek-V4-Pro vs Doubao-Seed-2.0-Pro | 46 | 11.25 (7.79-14.51) | 12.08 (7.83-15.49) | 0.815 (0.628-0.945) | <0.001 |
| FRES | DeepSeek-V4-Pro vs Gemini 3.5 Flash | 46 | 16.14 (12.06-20.45) | 12.37 (9.64-18.29) | 0.920 (0.796-0.994) | <0.001 |
| FRES | DeepSeek-V4-Pro vs Qwen3.7-Plus | 46 | 7.12 (4.30-9.88) | 7.69 (4.24-9.76) | 0.700 (0.462-0.891) | <0.001 |
| FRES | Doubao-Seed-2.0-Pro vs Gemini 3.5 Flash | 46 | 4.89 (0.22-9.60) | 2.89 (-2.82-8.58) | 0.252 (-0.082-0.541) | 0.153 |
| FRES | Doubao-Seed-2.0-Pro vs Qwen3.7-Plus | 46 | -4.13 (-7.35--0.74) | -4.24 (-9.59--1.54) | -0.421 (-0.689--0.114) | 0.016 |
| FRES | Gemini 3.5 Flash vs Qwen3.7-Plus | 46 | -9.02 (-13.50--4.85) | -7.31 (-11.18--2.32) | -0.593 (-0.819--0.327) | <0.001 |

***Note:*** *Pairwise comparisons used paired Wilcoxon signed-rank tests. Benjamini-Hochberg correction was applied separately to the 10 comparisons for each outcome. Differences are calculated as the first-listed interface minus the second-listed interface; positive rank-biserial correlations therefore favour the first-listed interface. Difference estimates and rank-biserial correlations are reported with 3,000-resample paired bootstrap 95% CIs. For ARI, CL, FKGL, GFI, and SMOG, lower values indicate easier readability; for FRES, higher values indicate easier readability.*
