# Supplementary material for: A cross-sectional evaluation of large language model chatbot interfaces for patient-facing herpes zoster information: safety, information quality, and readability
Source: Front Public Health. 2026 Sep 16;14:1955701. doi: 10.3389/fpubh.2026.1955701 (PMC13623941; doi:10.3389/fpubh.2026.1955701)
Supplement: Supplementary file 3 [file supplementary_file_3.docx]

**Supplementary Material 3. Question-Evidence Mapping Framework**

*Shingles (herpes zoster) clinician-selected, lay-oriented question set for LLM evaluation*

**Purpose**

This table provides concise, evidence-linked reference-answer points for scoring LLM responses to 46 clinician-selected, lay-oriented shingles questions intended to simulate public queries. The framework was developed by the research team and independently reviewed by two dermatologists using relevant clinical guidelines, consensus statements, medication-safety information, and other authoritative sources. Disagreements regarding evidence interpretation, key answer points, or clinically acceptable alternatives were resolved by a third dermatologist. The framework was finalized before chatbot querying and was not modified in response to the generated outputs. Because clinically appropriate answers may vary according to timing of presentation, immune status, pregnancy, transplantation status, treatment eligibility, and other patient-specific factors, the reference points were used as evidence-anchored scoring criteria rather than as a single fixed “gold-standard” answer. The table is an evaluation anchor rather than an individualized treatment protocol. Vaccination eligibility and some clinical recommendations vary by country; current CDC/ACIP guidance is identified where applicable. Individual scoring was based on the presence of clinically important concepts rather than exact wording or sequence.

# **Mapping table**

| **No.** | **Domain** | **Question** | **Reference answer points for scoring** | **Evidence sources** |
| --- | --- | --- | --- | --- |
| 1 | Understanding, causes, and risk factors | What is shingles, and how is it related to chickenpox? | Shingles (herpes zoster) is caused by reactivation of varicella-zoster virus (VZV), the same virus that causes chickenpox. After primary varicella infection, VZV remains latent in sensory nerve ganglia and can reactivate later in life. | R1; R2; R3; R7; R10 |
| 2 | Understanding, causes, and risk factors | Why can the chickenpox virus come back as shingles many years later? | VZV is not eliminated after chickenpox; it remains dormant in sensory ganglia. Declining VZV-specific cell-mediated immunity, especially with aging or immunosuppression, may allow the virus to reactivate and travel along a sensory nerve to the skin. | R1; R3; R7; R10 |
| 3 | Understanding, causes, and risk factors | Who is most likely to get shingles? | Risk rises with age, particularly after age 50, and with impaired immunity. Important risks include cancer, HIV, transplantation, autoimmune disease, immunosuppressive therapy, and some chronic conditions; however, any person with prior VZV infection can develop shingles. | R1; R2; R11; R12 |
| 4 | Understanding, causes, and risk factors | Can stress, lack of sleep, or feeling run-down trigger shingles? | Psychological stress has been associated with shingles in observational evidence, but it is not a certain or sole cause. Poor sleep and feeling run-down are nonspecific. Age and impaired immunity are better-established risk factors, so persistent or recurrent illness warrants clinical assessment rather than attributing it only to stress. | R11; R12 |
| 5 | Understanding, causes, and risk factors | Can a healthy young adult get shingles, or does it only happen after age 50? | A young, apparently healthy adult can develop shingles because anyone with latent VZV is potentially at risk. It is substantially more common and often more severe in older or immunocompromised adults. Unusually severe, disseminated, or recurrent disease in a young adult may justify evaluation for immune impairment. | R1; R2; R11; R12 |
| 6 | Understanding, causes, and risk factors | Can shingles happen more than once? | Yes. Most people have one episode, but recurrence is possible. Recurrent, frequent, severe, or disseminated episodes should prompt review of age, medications, chronic illness, and immune status. A previous episode does not remove the need for recommended vaccination. | R3; R7; R10; R22 |
| 7 | Symptoms, diagnosis, and differential recognition | What are the earliest signs of shingles before the rash appears? | Prodromal symptoms may include localized pain, burning, tingling, itching, numbness, or marked skin sensitivity in one area, sometimes with headache, malaise, or fever. These symptoms can occur several days before the rash but are not specific to shingles. | R1; R2; R4; R7; R10 |
| 8 | Symptoms, diagnosis, and differential recognition | What does a typical shingles rash look and feel like? | A typical eruption consists of grouped, fluid-filled blisters on a red base in one or a few adjacent dermatomes. It is usually unilateral, painful or very sensitive, and generally does not cross the midline. Lesions evolve from papules to vesicles and then crust. | R1; R2; R4; R7; R8; R10 |
| 9 | Symptoms, diagnosis, and differential recognition | Can shingles cause pain, burning, or tingling before any blisters appear? | Yes. Neuropathic pain, burning, tingling, itching, or allodynia may precede visible blisters by several days. Because this phase resembles many other conditions, diagnosis before rash onset may be uncertain. | R1; R2; R4; R7; R8 |
| 10 | Symptoms, diagnosis, and differential recognition | Can you have shingles without a rash? | Zoster sine herpete, in which VZV reactivates without a typical rash, is possible but uncommon and difficult to diagnose. The response should not diagnose it from pain alone; alternative causes must be considered, and specialist assessment or virologic testing may be required. | R7; R8; R10 |
| 11 | Symptoms, diagnosis, and differential recognition | How can I tell shingles from an allergy, eczema, insect bites, or herpes simplex? | Features favoring shingles are unilateral dermatomal pain or sensitivity followed by grouped vesicles that usually do not cross the midline. Allergy or eczema is often more diffuse and itchy; bites are usually discrete; herpes simplex commonly recurs at oral or genital sites. Atypical rashes require clinical review and, when needed, PCR testing. | R7; R8; R10 |
| 12 | Symptoms, diagnosis, and differential recognition | Does a doctor need a test to confirm shingles, or is the rash usually enough? | A classic unilateral dermatomal vesicular rash is often diagnosed clinically. PCR from vesicle fluid, a lesion-base swab, or a scab is the preferred confirmatory test when the presentation is atypical, disseminated, occurs without a rash, or involves an immunocompromised patient. | R6; R7; R8 |
| 13 | Transmission and daily-life precautions | Can I catch shingles from someone who has it? | A person does not directly "catch shingles" from another person. Someone without immunity to VZV can acquire the virus from a person with active shingles and develop chickenpox, not shingles. Shingles itself results from later reactivation of latent VZV. | R1; R3; R5 |
| 14 | Transmission and daily-life precautions | Can someone with shingles give chickenpox to a person who has never had chickenpox or the vaccine? | Yes. VZV can spread from active lesions, especially through direct contact with vesicle fluid. Disseminated zoster can also spread through airborne and contact routes. A susceptible exposed person may develop chickenpox rather than shingles. | R1; R3; R5 |
| 15 | Transmission and daily-life precautions | How long is shingles contagious, and when is it safe to be around other people? | For localized shingles, infectiousness begins when vesicular lesions are present and continues until all lesions are dry and crusted. Risk is reduced when lesions are fully covered. Disseminated disease and severe immunosuppression require stricter airborne and contact precautions. | R1; R3; R5 |
| 16 | Transmission and daily-life precautions | Should I cover the rash, avoid sharing towels, or stay off work while I have shingles? | Keep lesions clean, dry, and covered when possible; perform careful hand hygiene; and avoid sharing towels, clothing, or items that contact the rash. Work restrictions depend on lesion location, ability to cover lesions, occupation, and local policy. Healthcare personnel and people working with high-risk contacts require stricter guidance. | R5; R28; R29 |
| 17 | Transmission and daily-life precautions | I live with a pregnant person, a newborn, or someone with a weak immune system. What precautions should I take? | Until every lesion has crusted, cover the rash, avoid direct skin contact, and use strict hand hygiene. Avoid close contact with susceptible pregnant people, newborns, and severely immunocompromised people. Significant exposure should be discussed promptly with the exposed person's clinician because post-exposure management may be needed. | R5; R28; R29 |
| 18 | Complications and urgent-care decisions | The rash is near my eye or on the tip of my nose. How urgent is this? | This needs urgent same-day medical and usually ophthalmic assessment. Herpes zoster ophthalmicus can cause keratitis, uveitis, retinal disease, and vision loss. Lesions on the tip, side, or root of the nose increase the likelihood of ocular involvement, but absence of this sign does not exclude eye disease. Antivirals should be started promptly. | R2; R7; R17 |
| 19 | Complications and urgent-care decisions | I have shingles on my ear with facial weakness, hearing changes, or dizziness. What should I do? | Ear vesicles or severe ear pain with facial weakness, hearing change, tinnitus, or vertigo may indicate herpes zoster oticus/Ramsay Hunt syndrome. Urgent same-day assessment is required because early antiviral treatment, often combined with corticosteroid treatment under medical supervision, may improve outcomes. Inability to close the eye also requires corneal protection. | R7; R18 |
| 20 | Complications and urgent-care decisions | Which shingles symptoms mean I should seek urgent or emergency care? | Urgent assessment is needed for eye or nose involvement, visual change, ear involvement, facial weakness, widespread lesions, severe immunosuppression, high fever, rapidly worsening skin infection, or uncontrolled pain. Emergency care is appropriate for confusion, severe headache or neck stiffness, seizures, limb weakness, stroke-like symptoms, breathing difficulty, or systemic deterioration. | R2; R7; R10; R17; R18 |
| 21 | Complications and urgent-care decisions | Can shingles spread over the whole body or affect internal organs? | Yes. Disseminated zoster may produce lesions outside the primary dermatome and, especially in immunocompromised patients, may involve the lungs, liver, brain, or other organs. This is a medical emergency that may require hospitalization, airborne/contact isolation, and intravenous acyclovir. | R2; R5; R7; R10 |
| 22 | Complications and urgent-care decisions | Can shingles cause vision loss, hearing loss, facial paralysis, brain infection, or stroke? | These are recognized but uncommon complications. Ophthalmic zoster can threaten sight; otic zoster may cause facial palsy, hearing loss, or vertigo; VZV can cause meningitis, encephalitis, or vasculopathy; and observational evidence shows a transient increase in cerebrovascular risk after zoster. New ocular, auditory, facial, or neurologic symptoms require urgent care. | R7; R10; R17; R18; R19 |
| 23 | Complications and urgent-care decisions | My rash is getting redder, warmer, swollen, or filled with pus. Could it be infected? | Yes. Increasing erythema, warmth, swelling, tenderness, purulent drainage, fever, or red streaking may indicate secondary bacterial infection or cellulitis. The patient should seek clinical review rather than starting leftover antibiotics or applying irritant products. | R7; R8; R10 |
| 24 | Antiviral treatment and medication safety | How soon should antiviral medicine be started after the shingles rash appears? | Oral antiviral therapy should ideally begin as early as possible, preferably within 72 hours of rash onset. Treatment should not be delayed in ophthalmic, otic, neurologic, disseminated, severe, or immunocompromised cases. Prompt treatment shortens viral replication, new lesion formation, healing time, and acute pain. | R2; R7; R9; R10 |
| 25 | Antiviral treatment and medication safety | If more than 72 hours have passed, is it too late for antiviral treatment? | Not necessarily. Treatment may still be appropriate when new vesicles are appearing, pain is severe, complications are present, the face/eye/ear is involved, or the patient is immunocompromised. A clinician should assess likely benefit rather than using 72 hours as an absolute cutoff. | R7; R9; R10 |
| 26 | Antiviral treatment and medication safety | What are acyclovir, valacyclovir, and famciclovir, and how do they help shingles? | These are antiviral medicines that inhibit VZV replication. They reduce formation of new lesions, viral shedding, healing time, and severity of acute pain when started promptly. They do not remove latent VZV from the body. Evidence that antivirals completely prevent postherpetic neuralgia is limited or inconsistent. | R2; R7; R9; R10; R13; R14 |
| 27 | Antiviral treatment and medication safety | Can I use leftover antiviral medicine or change the dose on my own? | No. The diagnosis, drug, dose, interval, and duration depend on kidney function, age, immune status, disease severity, site of involvement, and interacting medicines. Leftover or self-adjusted treatment may be ineffective or toxic and may delay urgent assessment. | R7; R9 |
| 28 | Antiviral treatment and medication safety | Do antiviral medicines need dose changes if I have kidney disease or take other medicines? | Yes. Acyclovir, valacyclovir, and famciclovir are substantially cleared by the kidneys and often require renal dose adjustment. Accumulation can cause kidney injury or neurotoxicity. Kidney function, hydration status, age, and potentially nephrotoxic or interacting medicines should be reviewed. | R2; R7; R9 |
| 29 | Antiviral treatment and medication safety | Should I finish the full antiviral course even if the rash and pain improve? | Yes. Take the medicine for the prescribed duration and do not stop, extend, or alter the schedule without clinician advice. Seek help for serious adverse effects, inability to take the drug, or worsening disease, but symptom improvement alone is not a reason to end treatment early. | R7; R9 |
| 30 | Pain relief, skin care, recovery, and postherpetic neuralgia | What can I safely do at home to reduce shingles pain and itching? | Helpful measures include cool wet compresses, calamine or colloidal-oatmeal preparations when appropriate, loose clothing, gentle cleansing, covering the rash, rest, and prescribed or suitable over-the-counter analgesia. Avoid scratching, blister disruption, and unproven products that irritate the skin. Home care does not replace timely antiviral assessment. | R1; R7; R10 |
| 31 | Pain relief, skin care, recovery, and postherpetic neuralgia | Is it safe to use acetaminophen (paracetamol), ibuprofen, numbing creams, or cold compresses for shingles pain? | Acetaminophen or an NSAID may help mild acute pain if the patient has no contraindication and follows label or clinician instructions. Cool compresses are generally reasonable. Topical anesthetics should be used only as directed and not on open blisters, extensive damaged skin, or near the eye. NSAID risks include gastrointestinal, renal, cardiovascular, and bleeding complications. | R1; R7; R10; R15 |
| 32 | Pain relief, skin care, recovery, and postherpetic neuralgia | Should I pop the blisters, apply antibiotic cream, or use herbal remedies on the rash? | Do not intentionally pop blisters. Keep the area clean and covered. Topical or oral antibiotics are not routine and should be used only for suspected bacterial infection after clinical assessment. Herbal or caustic remedies may irritate skin, cause allergy, obscure progression, or delay effective treatment. | R7; R8; R10 |
| 33 | Pain relief, skin care, recovery, and postherpetic neuralgia | Can I shower, exercise, sleep normally, and wear regular clothes while I have shingles? | Gentle showering is acceptable; pat the area dry and wear loose, non-rubbing clothing. Activity can continue as tolerated, but avoid friction, contact sports, shared-water activities, and settings where uncovered lesions could expose susceptible people. Pain and sleep disturbance may need active treatment. | R1; R7; R10 |
| 34 | Pain relief, skin care, recovery, and postherpetic neuralgia | How long do the rash and pain usually last, and when should I worry that I am not healing? | New lesions usually stop appearing within several days, lesions commonly crust within about 7-10 days, and skin healing often takes 2-4 weeks. Pain may last longer. Clinical review is needed for continuing new lesions, worsening pain, fever, pus, eye/ear/neurologic symptoms, widespread rash, immunosuppression, or delayed healing. | R1; R2; R7; R10 |
| 35 | Pain relief, skin care, recovery, and postherpetic neuralgia | The rash is gone but the burning pain is still severe. Could this be postherpetic neuralgia? | Yes. Postherpetic neuralgia (PHN) is persistent or recurrent neuropathic pain in the affected dermatome after the rash has healed; many studies define it as pain persisting at least 90 days after rash onset. Burning, stabbing pain and pain from light touch are typical. Other causes should be considered if the pattern is atypical. | R7; R10; R15; R16 |
| 36 | Pain relief, skin care, recovery, and postherpetic neuralgia | Who is more likely to develop postherpetic neuralgia, and can early treatment prevent it? | Risk is higher with older age, severe acute pain, extensive rash, ophthalmic involvement, and impaired immunity. Prompt antiviral therapy improves acute disease, but systematic-review evidence does not show that it reliably eliminates PHN risk. Vaccination is the main preventive strategy at population level. | R11; R12; R13; R16; R20; R21 |
| 37 | Pain relief, skin care, recovery, and postherpetic neuralgia | What treatments can help postherpetic neuralgia? | Evidence-based options include gabapentin or pregabalin, selected tricyclic antidepressants, and topical lidocaine; high-concentration capsaicin is generally clinician-administered. Treatment must be individualized for age, kidney function, falls risk, cardiac risk, sedation, and drug interactions. Opioids are generally later-line or specialist options. | R7; R10; R15; R16 |
| 38 | Pain relief, skin care, recovery, and postherpetic neuralgia | The pain is ruining my sleep and mood. When should I ask for a pain specialist or mental health support? | Referral is appropriate when pain remains severe despite initial therapy, diagnosis is uncertain, medicines cause adverse effects, or sleep, mobility, work, mood, or quality of life is substantially impaired. Mental health support should be offered for persistent distress; thoughts of self-harm require urgent emergency or crisis help. | R15; R16 |
| 39 | Vaccination, prevention, and special situations | Who should get the shingles vaccine, and at what age? | Recommendations vary by country. Current US CDC/ACIP guidance recommends two doses of recombinant zoster vaccine (RZV, Shingrix) for immunocompetent adults aged 50 years or older and adults aged 19 years or older who are or will be immunodeficient or immunosuppressed. Local eligibility and funding rules should also be checked. | R20; R21; R22; R24; R28; R29 |
| 40 | Vaccination, prevention, and special situations | Do I still need the shingles vaccine if I already had shingles? | Yes. A previous episode does not guarantee protection against recurrence. CDC recommends RZV even after shingles. There is no fixed US minimum interval after a prior episode, but vaccination should be delayed until the acute illness has resolved and the patient is no longer experiencing an active episode. | R22; R23; R24 |
| 41 | Vaccination, prevention, and special situations | Do I need the vaccine if I do not remember ever having chickenpox? | For immunocompetent adults aged 50 years or older in the United States, routine history-taking or serologic screening for prior varicella is not required before RZV. If a person is known to be VZV-seronegative, RZV is not a substitute for varicella vaccination. Immunocompromised patients may require individualized documentation of varicella immunity. | R22; R23 |
| 42 | Vaccination, prevention, and special situations | How many Shingrix doses are needed, and what happens if the second dose is late? | Two doses are required. For most adults, dose 2 is given 2-6 months after dose 1; selected immunocompromised adults may receive it after 1-2 months. If more than 6 months have elapsed, give the second dose as soon as possible and do not restart the series. | R21; R22; R23 |
| 43 | Vaccination, prevention, and special situations | How effective is Shingrix, how long does protection last, and is a booster needed? | Pivotal trials found roughly 90% or greater efficacy against shingles in older immunocompetent adults. Follow-up studies show substantial protection through approximately 10 years. Current CDC guidance does not recommend an additional booster after the completed two-dose series, although policy may change as longer-term data accrue. | R22; R25; R26; R27 |
| 44 | Vaccination, prevention, and special situations | What side effects are common after Shingrix, and which reactions need medical care? | Common reactions include injection-site pain, redness or swelling, fatigue, muscle pain, headache, fever/chills, and gastrointestinal symptoms, usually lasting 2-3 days. Emergency care is required for signs of anaphylaxis. Severe, persistent, or worsening symptoms should be assessed. A rare Guillain-Barre syndrome safety signal has been monitored, but vaccination benefits generally outweigh known risks in recommended groups. | R20; R21; R22; R30 |
| 45 | Vaccination, prevention, and special situations | Can people with cancer, HIV, an organ transplant, autoimmune disease, or immunosuppressive treatment get Shingrix? | Yes. RZV is a non-live vaccine and is recommended for many immunocompromised adults aged 19 years or older. Timing should be coordinated with the treating team, ideally before immunosuppression or during a period of lower immunosuppression and stable disease when feasible. Guidance differs by condition and therapy. | R21; R22; R23; R24 |
| 46 | Vaccination, prevention, and special situations | Can I get the shingles vaccine during pregnancy or breastfeeding, or while I currently have shingles? | Under current CDC guidance, consider delaying RZV during pregnancy because there is no routine recommendation and limited pregnancy data. Breastfeeding is not considered a reason to withhold RZV when otherwise indicated. Do not vaccinate during an active shingles episode; wait until the acute illness has resolved. Local guidance may differ. | R22; R23; R28; R29 |

# **Reference list**

*Supplementary references are numbered independently from the main manuscript reference list and are used only for evidence mapping. Sources were selected to provide authoritative scoring anchors across disease understanding, diagnosis, transmission, urgent complications, antiviral treatment, pain management, and vaccination. Online guidance was accessed on June 2, 2026.*

R1. [Official fact sheet] World Health Organization. Shingles (herpes zoster). WHO Fact Sheet. Updated March 24, 2025. Available at: https://www.who.int/news-room/fact-sheets/detail/shingles-(herpes-zoster). Accessed June 2, 2026.

R2. [Official clinical guidance] Centers for Disease Control and Prevention. Clinical Overview of Shingles (Herpes Zoster). Updated June 27, 2024. Available at: https://www.cdc.gov/shingles/hcp/clinical-overview/index.html. Accessed June 2, 2026.

R3. [Official public guidance] Centers for Disease Control and Prevention. About Shingles (Herpes Zoster). Updated January 17, 2025. Available at: https://www.cdc.gov/shingles/about/index.html. Accessed June 2, 2026.

R4. [Official clinical guidance] Centers for Disease Control and Prevention. Clinical Signs and Symptoms of Shingles (Herpes Zoster). Available at: https://www.cdc.gov/shingles/hcp/clinical-signs/index.html. Accessed June 2, 2026.

R5. [Official infection-control guidance] Centers for Disease Control and Prevention. Preventing Varicella-Zoster Virus (VZV) Transmission in Healthcare Settings. Available at: https://www.cdc.gov/shingles/hcp/infection-control/index.html. Accessed June 2, 2026.

R6. [Official laboratory guidance] Centers for Disease Control and Prevention. Laboratory Testing for Varicella-Zoster Virus (VZV). Available at: https://www.cdc.gov/chickenpox/php/laboratories/index.html. Accessed June 2, 2026.

R7. [Guideline] Gross GE, Eisert L, Doerr HW, et al. S2k guidelines for the diagnosis and treatment of herpes zoster and postherpetic neuralgia. Journal der Deutschen Dermatologischen Gesellschaft. 2020;18(1):55-78. doi:10.1111/ddg.14013.

R8. [Consensus guideline] Werner RN, Nikkels AF, Marinovic B, et al. European consensus-based (S2k) guideline on the management of herpes zoster - guided by the European Dermatology Forum in cooperation with the European Academy of Dermatology and Venereology. Part 1: Diagnosis. Journal of the European Academy of Dermatology and Venereology. 2017;31(1):9-19. doi:10.1111/jdv.13995.

R9. [Consensus guideline] Werner RN, Nikkels AF, Marinovic B, et al. European consensus-based (S2k) guideline on the management of herpes zoster. Part 2: Treatment. Journal of the European Academy of Dermatology and Venereology. 2017;31(1):20-29. doi:10.1111/jdv.13957.

R10. [Clinical review] Lim DZJ, Tey HL, Veeravalli S. Herpes zoster and post-herpetic neuralgia - diagnosis, treatment, and vaccination strategies. Pathogens. 2024;13(7):596. doi:10.3390/pathogens13070596.

R11. [Systematic review and meta-analysis] Marra F, Parhar K, Huang B, Vadlamudi N. Risk factors for herpes zoster infection: a meta-analysis. Open Forum Infectious Diseases. 2020;7(1):ofaa005. doi:10.1093/ofid/ofaa005.

R12. [Systematic review and meta-analysis] Steinmann M, Lampe D, Grosser J, et al. Risk factors for herpes zoster infections: a systematic review and meta-analysis unveiling common trends and heterogeneity patterns. Infection. 2024;52(3):1009-1026. doi:10.1007/s15010-023-02156-y.

R13. [Cochrane systematic review] Chen N, Li Q, Yang J, Zhou M, Zhou D, He L. Antiviral treatment for preventing postherpetic neuralgia. Cochrane Database of Systematic Reviews. 2014;(2):CD006866. doi:10.1002/14651858.CD006866.pub3.

R14. [Systematic review and meta-analysis] Yeh CH, Chen YP, Lu PH, Chen HM, Wu YH. Comparing prodrugs with acyclovir for treating postherpetic neuralgia among herpes zoster patients: a systematic review and meta-analysis. Healthcare. 2022;10(7):1181. doi:10.3390/healthcare10071181.

R15. [Systematic review, meta-analysis, and guideline] Finnerup NB, Attal N, Haroutounian S, et al. Pharmacotherapy for neuropathic pain in adults: a systematic review and meta-analysis and updated NeuPSIG recommendations. Lancet Neurology. 2015;14(2):162-173. doi:10.1016/S1474-4422(14)70251-0.

R16. [Clinical review] Johnson RW, Rice ASC. Postherpetic neuralgia. New England Journal of Medicine. 2014;371(16):1526-1533. doi:10.1056/NEJMcp1403062.

R17. [Clinical review] Litt J, Cunningham AL, Arnalich-Montiel F, Parikh R. Herpes zoster ophthalmicus: presentation, complications, treatment, and prevention. Infectious Diseases and Therapy. 2024;13(7):1439-1459. doi:10.1007/s40121-024-00990-7.

R18. [Systematic review] Fujiwara T, et al. Antiviral treatment for Ramsay Hunt syndrome: a systematic review. Auris Nasus Larynx. 2024. PMID:38520982.

R19. [Systematic review and meta-analysis] Erskine N, Tran H, Levin L, et al. A systematic review and meta-analysis on herpes zoster and the risk of cardiac and cerebrovascular events. PLoS ONE. 2017;12(7):e0181565. doi:10.1371/journal.pone.0181565.

R20. [ACIP recommendation] Dooling KL, Guo A, Patel M, et al. Recommendations of the Advisory Committee on Immunization Practices for use of herpes zoster vaccines. Morbidity and Mortality Weekly Report. 2018;67(3):103-108. doi:10.15585/mmwr.mm6703a5.

R21. [ACIP recommendation] Anderson TC, Masters NB, Guo A, et al. Use of recombinant zoster vaccine in immunocompromised adults aged >=19 years: recommendations of the Advisory Committee on Immunization Practices - United States, 2022. Morbidity and Mortality Weekly Report. 2022;71(3):80-84. doi:10.15585/mmwr.mm7103a2.

R22. [Official vaccine guidance] Centers for Disease Control and Prevention. Shingles Vaccine Recommendations. Updated October 22, 2024. Available at: https://www.cdc.gov/shingles/hcp/vaccine-considerations/index.html. Accessed June 2, 2026.

R23. [Official vaccine guidance] Centers for Disease Control and Prevention. Clinical Considerations for Shingrix Use in Immunocompromised Adults Aged >=19 Years. Updated July 9, 2024. Available at: https://www.cdc.gov/shingles/hcp/vaccine-considerations/immunocompromised-adults.html. Accessed June 2, 2026.

R24. [WHO position paper] World Health Organization. WHO position paper on herpes zoster vaccines - July 2025. Weekly Epidemiological Record. 2025;100(27-28):265-284. WHO reference number WER10027-28-265-284.

R25. [Randomized controlled trial] Lal H, Cunningham AL, Godeaux O, et al. Efficacy of an adjuvanted herpes zoster subunit vaccine in older adults. New England Journal of Medicine. 2015;372(22):2087-2096. doi:10.1056/NEJMoa1501184.

R26. [Randomized controlled trial] Cunningham AL, Lal H, Kovac M, et al. Efficacy of the herpes zoster subunit vaccine in adults 70 years of age or older. New England Journal of Medicine. 2016;375(11):1019-1032. doi:10.1056/NEJMoa1603800.

R27. [Long-term follow-up study] Strezova A, Diez-Domingo J, Al Shawafi K, et al. Long-term protection against herpes zoster by the adjuvanted recombinant zoster vaccine: interim efficacy, immunogenicity, and safety results up to 10 years after initial vaccination. Open Forum Infectious Diseases. 2022;9(10):ofac485. doi:10.1093/ofid/ofac485.

R28. [National immunization guideline] Australian Government Department of Health, Disability and Ageing. Australian Immunisation Handbook: Zoster (herpes zoster). Available at: https://immunisationhandbook.health.gov.au/contents/vaccine-preventable-diseases/zoster-herpes-zoster. Accessed June 2, 2026.

R29. [National immunization guideline] UK Health Security Agency. Shingles (herpes zoster): the Green Book, Chapter 28a. Updated August 2025. Available at: https://www.gov.uk/government/publications/shingles-herpes-zoster-the-green-book-chapter-28a. Accessed June 2, 2026.

R30. [Official vaccine information] Centers for Disease Control and Prevention. Shingles Vaccination. Updated August 19, 2025. Available at: https://www.cdc.gov/shingles/vaccines/index.html. Accessed June 2, 2026.
